# Machine Learning and Deep Learning Techniques for Prediction and Diagnosis of Leptospirosis: Systematic Literature Review

**DOI:** 10.2196/67859

**Published:** 2025-05-29

**Authors:** Suhila Sawesi, Arya Jadhav, Bushra Rashrash

**Affiliations:** 1 Health Informatics and Bioinformatics Program, College Of Computing, Grand Valley State University, 333 Michigan St. NE, Grand Rapids, MI, 49503, United States, 1 616-331-7827 ext 17827; 2 Data Science, College Of Computing, Grand Valley State University, Allendale, MI, United States; 3 Department of Biomedical Science, College of Liberal Arts and Sciences, Grand Valley State University, Allendale, MI, United States

**Keywords:** leptospirosis, machine learning, deep learning, prediction models, diagnosis, artificial intelligence, convolutional neural networks, support vector machines, transfer learning, zoonotic diseases

## Abstract

**Background:**

Leptospirosis, a zoonotic disease caused by *Leptospira* bacteria, continues to pose significant public health risks, particularly in tropical and subtropical regions.

**Objective:**

This systematic review aimed to evaluate the application of machine learning (ML) and deep learning (DL) techniques in predicting and diagnosing leptospirosis, focusing on the most used algorithms, validation methods, data types, and performance metrics.

**Methods:**

Using Preferred Reporting Items for Systematic Reviews and Meta-Analyses (PRISMA) guidelines, Checklist for Critical Appraisal and Data Extraction for Systematic Reviews of Prediction Modelling Studies (CHARMS), and Prediction model Risk of Bias Assessment Tool (PROBAST) tools, we conducted a comprehensive review of studies applying ML and DL models for leptospirosis detection and prediction, examining algorithm performance, data sources, and validation approaches.

**Results:**

Out of a total of 374 articles screened, 17 studies were included in the qualitative synthesis, representing approximately 4.5% of the initial pool. The review identified frequent use of algorithms such as support vector machines, artificial neural networks, decision trees, and convolutional neural networks (CNNs). Among the included studies, 88% (15/17) used traditional ML methods, and 24% (4/17) used DL techniques. Several models demonstrated high predictive performance, with reported accuracy rates ranging from 80% to 98%, notably with the U-Net CNN achieving 98.02% accuracy. However, public datasets were underused, with only 35% (6/17) of studies incorporating publicly available data sources; the majority (65%, 11/17) relied primarily on private datasets from hospitals, clinical records, or regional surveillance systems.

**Conclusions:**

ML and DL techniques demonstrate potential for improving leptospirosis prediction and diagnosis, but future research should focus on using larger, more diverse datasets, adopting transfer learning strategies, and integrating advanced ensemble and validation techniques to strengthen model accuracy and generalization.

## Introduction

### Overview of Leptospirosis and Its Diagnosis

Leptospirosis, a zoonotic disease caused by pathogenic *Leptospira* bacteria, is a global public health concern, with an estimated 1.03 million cases and approximately 58,900 deaths annually [1]. The disease is particularly prevalent in tropical and subtropical regions, where environmental factors such as heavy rainfall, poor sanitation, and frequent flooding facilitate bacterial survival and transmission. Although less common, leptospirosis also occurs in temperate regions, including the United States, especially in areas prone to flooding or with high populations of animal carriers like rodents and livestock [1].

In the United States, the Centers for Disease Control and Prevention (CDC) reports approximately 100‐150 cases annually, with the majority occurring in Puerto Rico and Hawaii due to their specific environmental conditions. Isolated cases in areas like New York City and California highlight the mobility of the disease and its potential for travel-related transmission [1].

Transmission primarily occurs through direct contact with the urine or reproductive fluids of infected animals or exposure to contaminated water and soil, especially following periods of heavy rainfall [2]. High-risk activities include wading, swimming, or boating in potentially contaminated freshwater. Effective control measures include improving sanitation, controlling rodent populations, and educating at-risk populations. The disease manifests with a broad range of clinical symptoms, from mild flu-like symptoms to severe complications such as Weil’s disease, characterized by jaundice, renal failure, pulmonary hemorrhage, and multi-organ dysfunction, which can lead to death if not treated promptly [1].

The diagnosis of leptospirosis is challenging due to the nonspecific nature of its early symptoms, which often overlap with other febrile illnesses. Traditional diagnostic methods include the microscopic agglutination test (MAT), considered the gold standard, and polymerase chain reaction (PCR). MAT is labor-intensive and requires specialized laboratory capabilities, making it less accessible in many endemic regions [2]. PCR, while offering early detection by identifying *Leptospira* DNA in blood or urine, also requires advanced laboratory infrastructure. Rapid diagnostic tests (RDTs) provide quicker results, but their sensitivity and specificity can vary depending on the *Leptospilora* serovars and disease stages, limiting their effectiveness in some settings [2,3].

### Machine Learning and Deep Learning in Disease Detection

Machine learning (ML) and deep learning (DL) have emerged as powerful tools in the field of disease detection and management. ML involves training computers to apply past experiences to solve new problems, leveraging algorithms that enable the machine to identify patterns, make predictions, and produce insightful judgments based on data. The increasing availability of computational power and data storage has significantly boosted the application of ML across various fields, including public health. In the context of infectious diseases like leptospirosis, ML can analyze large datasets, including clinical and laboratory data, to identify patterns and relationships that might not be apparent through traditional statistical methods [4-6].

DL, a subset of ML, further enhances these capabilities by using neural networks with multiple layers to automatically extract, analyze, and understand useful information from raw data. Unlike traditional ML techniques that rely on handcrafted features, DL models are capable of automatic feature engineering, which significantly enhances classification performance. DL techniques, driven by neural networks, are known for their accuracy and performance, particularly in complex tasks such as image recognition and analysis [6-8]. For example, convolutional neural networks (CNNs) have been successfully applied to medical imaging, enabling the precise identification and classification of pathogens in microscopy images [9], which is crucial for diseases like leptospirosis.

The application of ML and DL in leptospirosis diagnosis represents a significant advancement over traditional methods. ML algorithms can analyze clinical and laboratory data, including patient symptoms, demographic information, and test results, to predict the likelihood of leptospirosis. This capability is particularly valuable in settings where access to advanced diagnostics is limited, as it allows for earlier and more accurate detection, potentially reducing the time to diagnosis and improving patient outcomes [10].

DL models, particularly CNNs, have shown great promise in analyzing blood and urine samples, medical imaging, and environmental data to predict the presence of *Leptospira* or the likelihood of an outbreak. These models can distinguish *Leptospira* bacteria in microscopy images with high accuracy, reducing the need for skilled microbiologists and improving diagnostic accessibility in low-resource settings [4].

One of the most significant advantages of using ML and DL in leptospirosis diagnosis is their ability to integrate diverse data types—such as clinical, laboratory, and environmental data—into comprehensive predictive models. These models can be used for individual patient diagnosis and public health surveillance, enabling more targeted and timely interventions. For example, predictive models that incorporate climatic and environmental factors, such as rainfall patterns and flooding data, can help identify regions at higher risk for leptospirosis outbreaks, allowing for proactive disease control measures [10].

Despite the significant potential of ML and DL to revolutionize the diagnosis and management of leptospirosis, comprehensive reviews focusing specifically on their application in this area are scarce. Most existing reviews have primarily concentrated on more prevalent conditions such as tuberculosis, malaria, and COVID-19, with minimal attention given to zoonotic diseases like leptospirosis [4,5]. Furthermore, there is a recognized gap in the literature concerning the application of advanced AI techniques in the context of neglected tropical diseases, such as leptospirosis, where the potential for these technologies to improve diagnostic accuracy remains underexplored [6,11]. This gap underscores the need for a focused review that synthesizes current research, identifies the most effective ML and DL models, and evaluates their impact on public health outcomes related to leptospirosis.

The aim of this systematic review is to comprehensively evaluate the application of ML and DL techniques in the prediction and diagnosis of leptospirosis. This review will address the following key research questions:

1. Which ML and DL algorithms are most frequently used in leptospirosis prediction and diagnosis, and how well do they perform?
2. What validation methods are most used in the evaluation of ML and DL models for leptospirosis? and how reliable are these methods?
3. What types of data are most used in ML and DL models for leptospirosis? How does the type of data influence the performance of these models?
4. What are the main challenges and limitations identified in the research studies regarding ML and DL applications in leptospirosis prediction and diagnosis?

## Methods

### Study Design

We conducted a systematic review following the Preferred Reporting Items for Systematic Reviews and Meta-Analyses (PRISMA) checklist [12] ( Checklist 1). The Checklist for Critical Appraisal and Data Extraction for Systematic Reviews of Prediction Modelling Studies (CHARMS) was used to frame this review’s objectives [13].

#### Search Strategy

PubMed, IEEE, ACM, and Web of Science databases were searched for articles published from inception till May 29, 2024. Hand-searching of references within included articles was conducted to shortlist other potential articles. Our search strategy used a combination of subject terms related to “machine learning” and “Leptospirosis” (see Multimedia Appendix 1).

#### Eligibility Criteria

We included full-text English language articles that developed or validated diagnostic or predictive ML models for human leptospirosis. Our review focused specifically on ML and DL methods, including logistic regression, Bayesian learning, and generalized additive models when these were implemented within an ML or DL framework [14].

Several categories of studies were excluded. First, we omitted case reports, case series, letters, corrigenda, editorial commentaries, literature reviews, and meta-analyses. Second, we excluded purely applied statistical methods that were not integrated with ML or DL frameworks, including traditional statistical analyses that did not incorporate ML optimization techniques. Third, non-artificial intelligence methods as well as general artificial intelligence (AI) approaches that could not be classified as either ML or DL (such as rule-based expert systems without learning components or symbolic AI methods) were excluded [14].

The distinction between included and excluded methods was based on whether the approach involved automated learning from data. For instance, while standard logistic regression was excluded, logistic regression implemented with ML techniques like automated feature selection or hyperparameter tuning was included. Similarly, simple threshold-based diagnostic rules were excluded unless they were derived through ML processes. This approach ensured our review focused specifically on applications of ML and DL technologies in leptospirosis diagnosis and prediction.

In this review, diagnostic ML models refer to models that predict the disease status of an individual, while predictive models forecast the probability of future occurrence of the disease in an individual.

### Study Selection

A total of 3 independent reviewers (SS, AJ, and BR) conducted the initial search across 4 databases using predefined search terms within the title and abstract, strictly following the inclusion and exclusion criteria. Zotero bibliography software was used to manage the search results by tracking reasons for inclusion and exclusion, grouping records, importing PDFs, and exporting data to Microsoft Excel for extraction. The interrater agreement between the coauthors was evaluated using Cohen’s kappa (κ>0.80) [15,16], ensuring a high level of consistency across the reviewers.

To enhance the screening process, we employed ChatGPT-4o (June 2024 version) as a fourth reviewer. This advanced large language model (LLM) was specifically selected for its proven capabilities in biomedical text analysis (OpenAI) and superior handling of technical medical terminology compared to previous versions. We opted to use only this single model to maintain consistency in evaluation criteria and avoid potential variability from multiple LLMs. During implementation, abstracts were systematically input to ChatGPT-4o with standardized prompts mirroring our inclusion and exclusion criteria, and all outputs were automatically logged for verification.

While ChatGPT-4o provided valuable preliminary classifications (achieving 88% initial alignment with human reviewers in our pilot test), we implemented a rigorous 3-stage human verification protocol: (1) initial matching of AI recommendations with human decisions, (2) consensus discussion for discrepancies (κ<0.80), and (3) final unanimous approval. For example, the model initially recommended including 12 statistical modeling studies that were properly excluded after human review. This AI-assisted process reduced initial screening time by 30% while maintaining 100% alignment with final human decisions through our verification protocol, which followed PRISMA-AI guidelines [12] to mitigate potential AI limitations.

We emphasize that ChatGPT-4o served strictly in an advisory capacity, and no studies were included solely based on its recommendation. This approach aligns with emerging best practices for LLM-assisted systematic reviews [17,18], with complete verification records available in our supplementary materials (Multimedia Appendix 2).

### Quality Assessment

The articles selected for inclusion were then subjected to a quality assessment using the Prediction model Risk of Bias Assessment Tool (PROBAST) tool [13], which categorizes bias as low, medium, or high (see Multimedia Appendix 3). All authors assessed the quality of their respective parts, starting with a pilot of 5 articles to ensure consistency. The PROBAST tool evaluates risk of bias based on 4 segments—participants, predictors, outcome, and analysis. Each segment’s risk of bias was rated as high, medium, low, or unclear. If any domain suggested a high risk of bias, the overall risk of bias for that study was considered high. These studies were not excluded but were analyzed to understand their limitations and impact on the overall findings. The assessment was conducted independently by all authors, ensuring a thorough evaluation process.

### Data Extraction

For the extraction process, a standardized form was used to collect data relevant to the review’s objectives. This form was adapted from the CHARMS [15] and the Transparent Reporting of a Multivariable Prediction Model for Individual Prognosis or Diagnosis (TRIPOD) guidelines [19]. The extracted information included publication type, publication year, author, title, country of research, source of data, type of data (public or private), overall number of samples, and data collection methods (see Multimedia Appendix 4).

Additional details were gathered on the ML and DL algorithms used, including whether the models were pretrained or developed from scratch, as well as the use of transfer learning, data augmentation, validation methods, and evaluation metrics. The strength of leptospirosis predictions was documented using performance metrics. Tasks were categorized into segmentation, classification, and object detection, noting the type of classification and any limitations.

To ensure consistency, reviewers conducted a pilot phase where they independently extracted data from the first 5 articles and compared results, achieving a high agreement (κ=0.98). Afterward, all 17 articles were reviewed, and discrepancies were resolved through discussion. The studies were then rigorously categorized before moving to theme formation. One author (SS) defined the themes, which were reviewed and adjusted by the other authors (AJ and BR) to ensure comprehensive categorization.

### Outcomes Assessed

The primary outcomes assessed in this review include the diagnostic and predictive performance of various ML and DL methods for leptospirosis detection, focusing on metrics like accuracy, area under the curve (AUC), sensitivity, and specificity. It also evaluates the applicability and generalizability of these models in health care settings, emphasizing the integration of advanced neural network architectures, transfer learning, and data augmentation to enhance performance.

### Data Analysis

We grouped the collected studies into summary tables based on the type of ML and DL models used for leptospirosis detection. R (version 4.3.2; R Foundation for Statistical Computing) was used to perform both descriptive statistical analyses and create visualizations.

## Results

### Search and Selection Results

**Figure 1.**
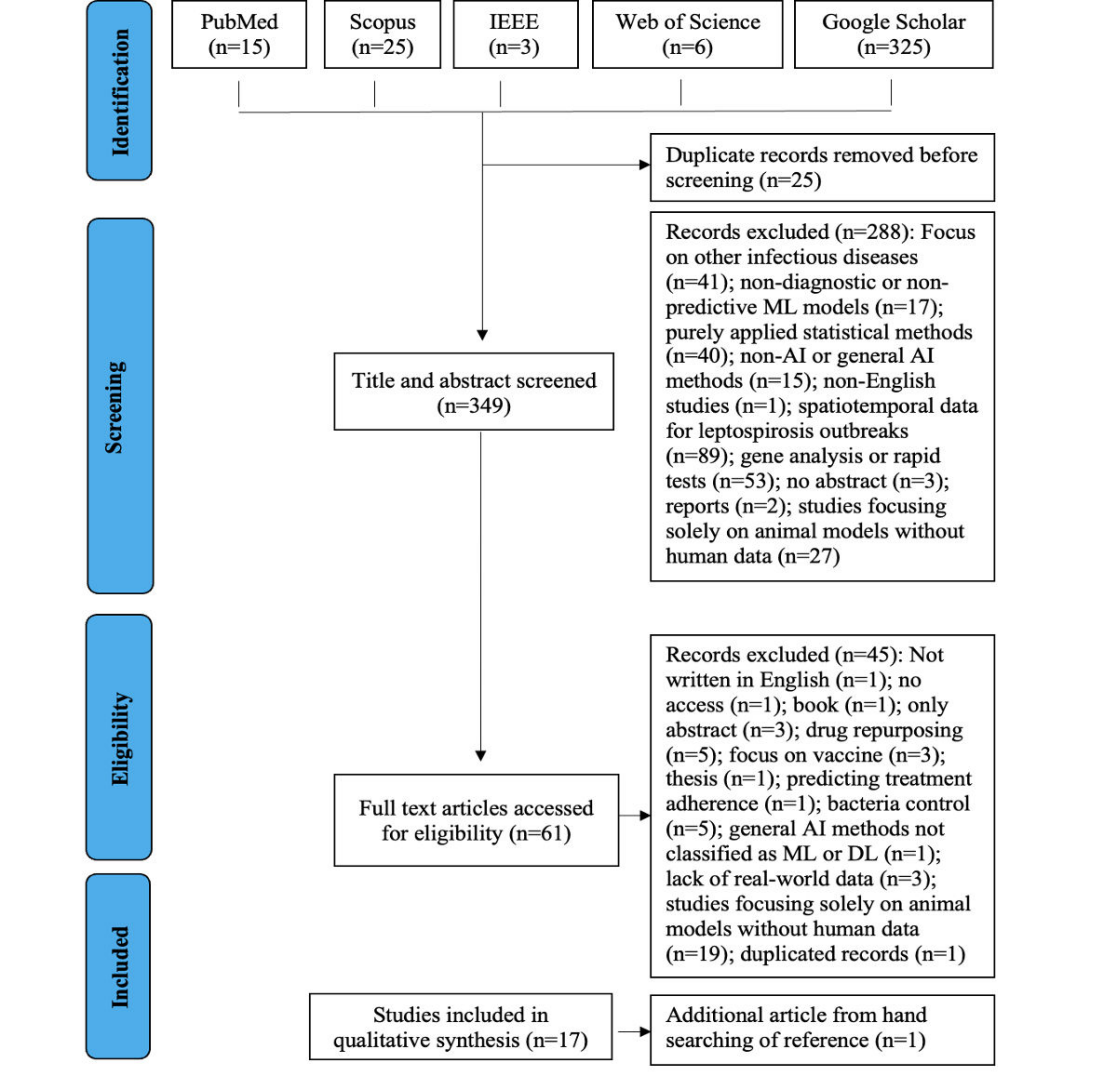
Figure 1. PRISMA (Preferred Reporting Items for Systematic Reviews and Meta-Analyses) flow diagram illustrating the search and selection process used to identify relevant studies. AI: artificial intelligence; ML: machine learning; DL: deep learning.

Figure 1 illustrates the process of identifying relevant literature. A comprehensive search across 5 databases yielded a total of 374 articles. After removing 25 duplicate records, 349 unique articles were screened based on their titles and abstracts. Following this initial screening, 61 articles were selected for full-text review. Of these, 45 were excluded for various reasons, including not meeting inclusion criteria or insufficient data for analysis. Ultimately, 16 studies were included in the review, with 1 additional study identified through hand-searching reference lists, bringing the total to 17 studies included in the qualitative synthesis.

### Study Description

**Figure 2.**
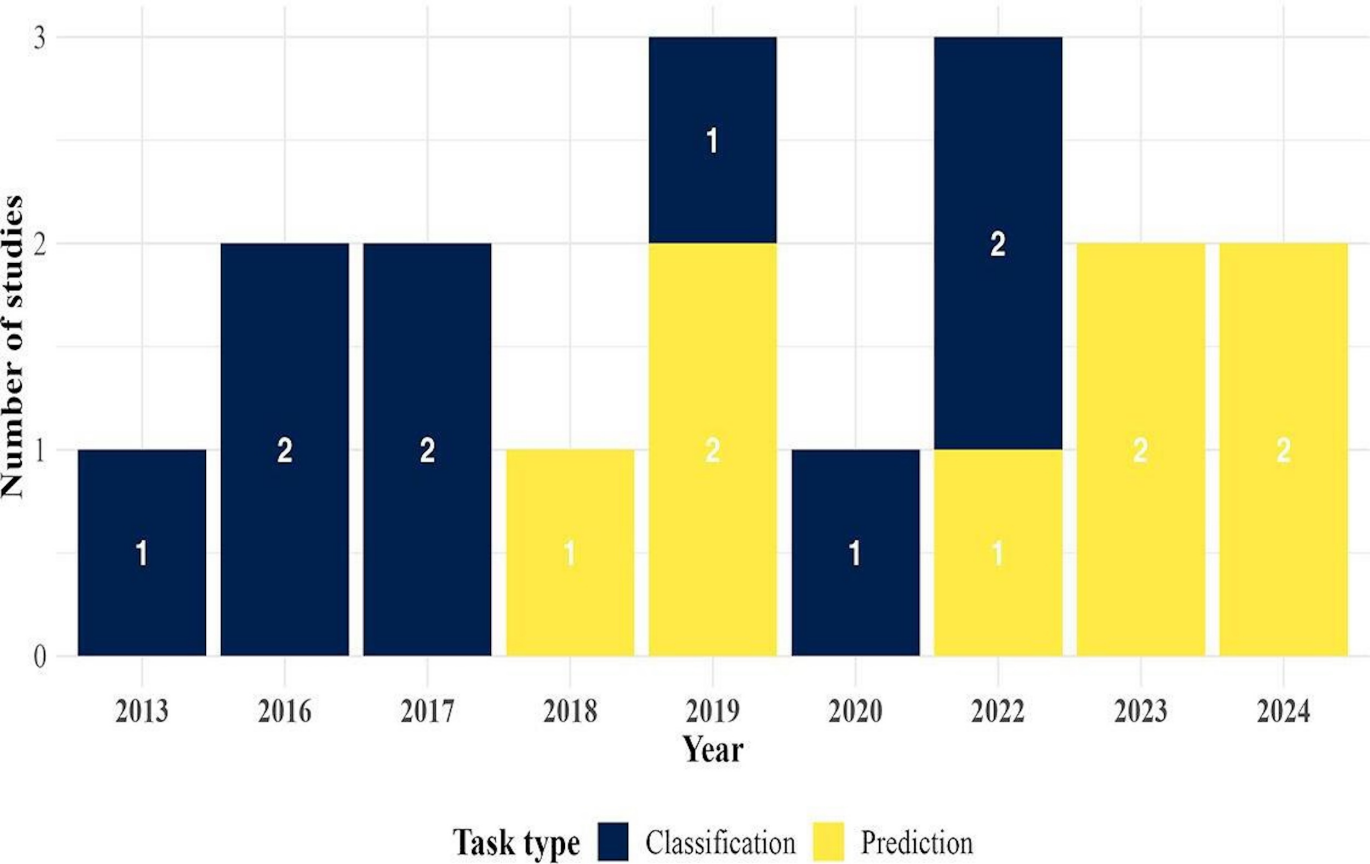
Distribution of studies on machine learning and deep learning applications for leptospirosis diagnosis and prediction by year and task type.

In this systematic review, we examined 17 studies (n=17) published between 2012 and 2024 that used ML and DL techniques for the prediction and diagnosis of leptospirosis (see Figure 2). Most studies were published in 2019 (n=3, 18%), 2022 (n=3, 18%), and 2023 (n=3, 18%). Brazil was the most common country of research, contributing 4 studies (24%), followed by New Caledonia with 3 studies (18%). Regarding data sources, 6 studies (35%) used health records, 6 studies (35%) used environmental data, and 5 studies (29%) used epidemiological data.

Most studies (11/17, 65%) focused on predictive modeling, while 6/17 studies (35%) concentrated on diagnosis. ML algorithms were overwhelmingly preferred, with 15/17 studies (88%) using techniques such as SVM, decision tree (DT), and random forests. DL algorithms, including CNN and multilayer perceptrons (MLPs), were used in 4/17 studies (24%), and only 1/17 study (6%) combined both ML and DL methods. All studies developed models from scratch without using transfer learning, and only 1/17 study (6%) reported the application of data augmentation techniques.

Regarding model validation, cross-validation methods were most frequently used in 11/17 studies (65%), while holdout validation methods, such as train and test splits, were used in 6/17 studies (35%).

### Assessment of Risk of Bias in Machine Learning Models

**Figure 3.**
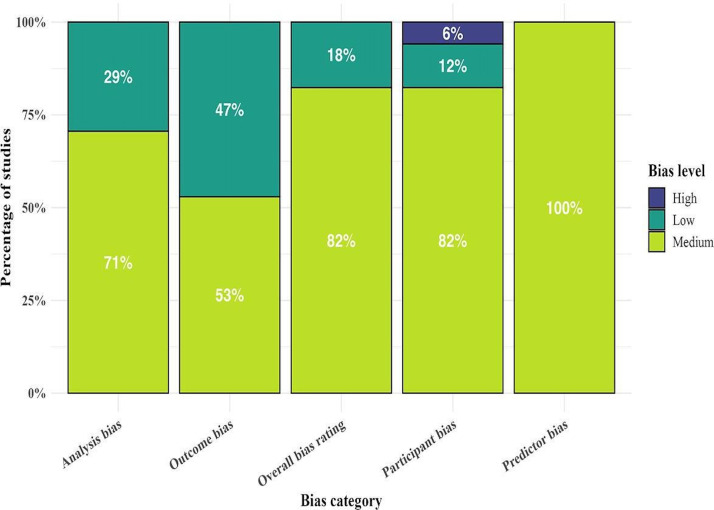
Distribution of risk of bias across domains in machine learning and deep learning studies for leptospirosis.

In assessing the risk of bias across the included 17 studies, most were categorized as having a medium risk across key domains (Figure 3). A total of 14 studies (82%) were rated as having a medium risk of bias related to participant selection, primarily due to the selection of specific regions or populations that may not fully represent broader leptospirosis cases. Examples include studies by [10,20-32]. In addition, 2 studies (12%) [33,34] were rated as low risk, while 1 study (6%) [35] was rated as high risk due to narrower participant selection.

Regarding predictors, 16 studies (94%) demonstrated a medium risk of bias, often because they relied heavily on environmental or clinical data without fully accounting for confounding variables. Only 1 study (6%) [34] was rated as low risk in this domain. For outcome bias, 13 studies (76%) [10,20-27,29,30,34,35] were assessed as low risk, with clear and consistent definitions applied across participants. A total of 4 studies (24%) [28,31-33] exhibited medium risk, mainly due to subjective outcome determinations or a lack of standardized measures.

In the analysis domain, 13 studies (76%) [10,22-33] demonstrated medium risk due to concerns about validation techniques, handling of missing data, and small sample sizes, while 4 studies (24%) [20,21,34,35] were rated as low risk, reflecting stronger analytical methodologies.

### Frequently Used ML and DL Algorithms for Leptospirosis Prediction and Diagnosis

**Figure 4.**
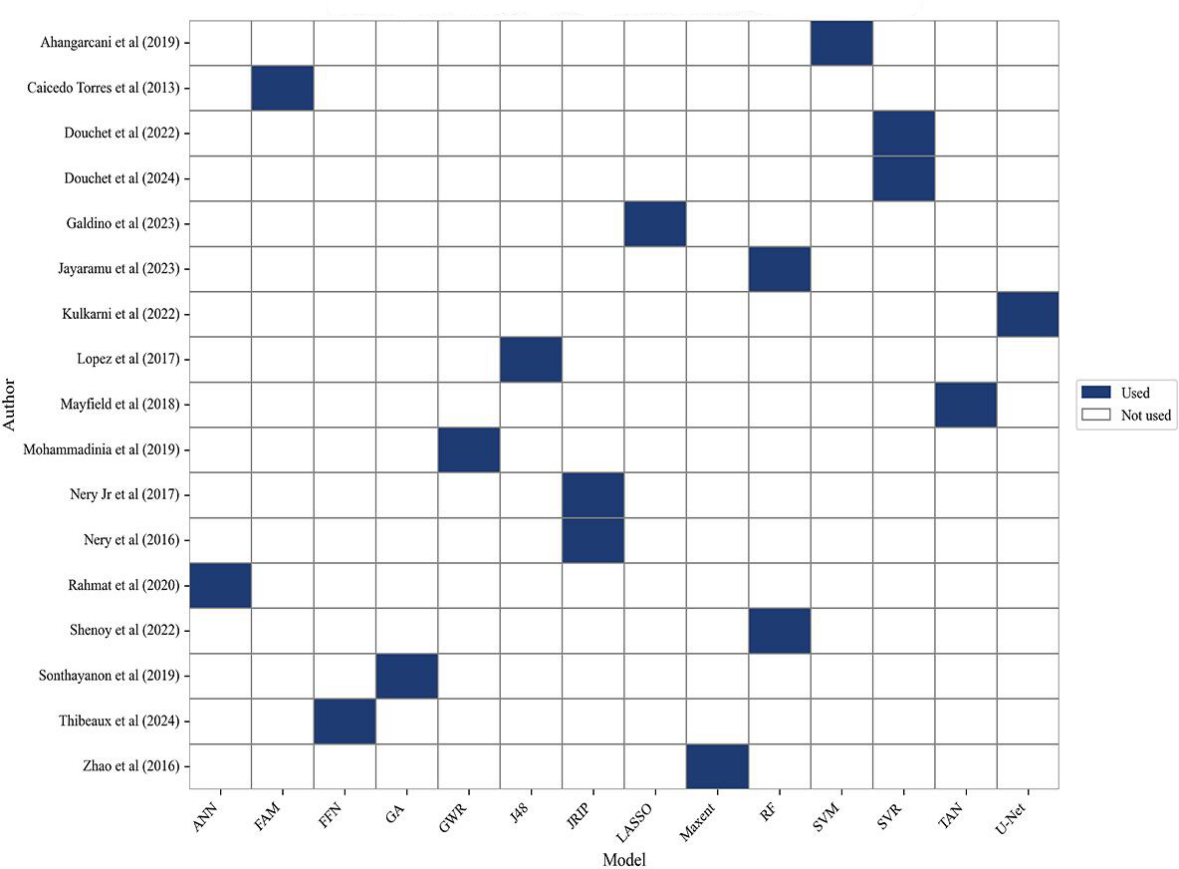
Heatmap of classifier usage across included studies [10,20-35]. ANN: artificial neural network; DL: deep learning; FAM: fuzzy adaptive resonance theory mapping; FFN: feed forward network; GA: genetic algorithm; GWR: geographically weighted regression; J48: J48 decision tree; JRIP: repeated incremental pruning to produce error reduction; LASSO: least absolute shrinkage and selection operator regression; Maxent: maximum entropy model; ML: machine learning; RF: random forest; SVM: support vector machine; SVR: support vector regression; TAN: tree augmented naïve network; U-Net: U-Net convolutional neural network.

This systematic review considered all ML and DL techniques used in the included studies and examined their applications in either leptospirosis prediction or diagnosis (classification). Figure 4 illustrates the distribution of classifiers across the studies, highlighting the diversity of approaches and the frequency of use of certain models.

For prediction tasks, the most commonly used ML technique was support vector regression (SVR), applied in 2 studies [32,33]. In addition to SVR, several other ML techniques were prominently featured. These included the naïve networks and TAN (tree augmented naive) networks used by Mayfield et al [24] for predictive risk mapping, and the random forest classifier and M1 mixed model used by Jayaramu et al [30] for predictive risk modeling.

Models like LeptoScore and QuickLepto, applied by Galdino et al [10], further illustrate the diversity of approaches taken in predictive modeling. SVM and MLP used in predictive risk modeling by Ahangarcani et al [26], while Mohammadinia et al [27] applied geographically weighted regression, generalized linear models, artificial neural networks (ANNs), and SVM for similar tasks. In addition, feedforward neural networks were used by [31] in prediction tasks, demonstrating the growing role of neural network models in this domain.

For diagnosis (classification) tasks, ANNs were widely used, appearing in studies by [20,28]. FuzzyARTMAP and ARTMAP-IC, both variants of ANN, were also used by [20] to achieve classification. In addition, Bayesian classifiers such as Naïve Bayes were applied by [21,29], further showcasing the diversity of ML techniques in classification tasks.

CNNs were another frequently used DL model for classification. Specifically, U-Net, a variant of CNN, was used by [34] and achieved an impressive accuracy of 98%. Other classifiers, such as k-nearest neighbors (KNNs), DTs (J48), and random forests, were used in multiple studies, with [23,29] demonstrating their efficacy in disease classification.

Performance evaluations showed that many studies combined multiple classifiers. For example [29], applied naïve Bayes, KNN, MLP, J48 decision tree, random forest, multinomial logistic regression, and Adaboost within the same study, with random forest achieving the highest performance at 87% accuracy and 91% sensitivity.

Hybrid approaches combining ML and DL were also present, such as the use of a genetic algorithm combined with both ML and DL techniques in [25], which attained an accuracy of 99%.

Upon comparing prediction and diagnosis (classification) tasks, it becomes evident that ML models dominated predictive risk mapping studies, while DL models, particularly U-Net, were more frequently used in classification tasks related to disease detection. For risk modeling, techniques like decision trees (J48) and random forest classifiers were commonly applied, with high specificity rates achieved in studies such as those by [21,30].

Interestingly, 50% of the studies (n=8) used more than one algorithm to assess performance, highlighting the importance of comparative evaluations in the field. Supervised-learning algorithms were predominant throughout the studies, with no mention of unsupervised-learning methods, such as K-means, or reinforcement-learning algorithms.

In summary, the most frequent algorithms in prediction tasks were SVR and random forest, while in diagnosis, ANN and U-Net stood out as the most frequently used. Across both types of tasks, the performance metrics indicated high accuracy and sensitivity, showcasing the reliability of ML and DL techniques in leptospirosis research.

### Algorithm Performance Metrics

Performance assessment is a crucial process in evaluating ML and DL models. Various metrics are used to measure model performance, including accuracy, sensitivity, specificity, precision, *F*_1_-score, AUC, mean squared error (MSE), *R*-squared (*R*²), mean absolute error (MAE), and root mean squared error (RMSE). These metrics are typically evaluated using hidden or unseen examples to assess model generalizability. In the included studies, accuracy was the most frequently reported metric, followed by sensitivity, precision, specificity, and *F*_1_-score. For prediction tasks, MSE, MAE, RMSE, and *R*² were used to assess regression performance.

Tables1  2 demonstrates the widespread use of accuracy rates across various models. The studies examined used accuracy as the primary indicator of performance, although other metrics such as sensitivity and specificity were also highlighted. For instance, the performance of the ANN in the Seremban City dataset reached 80% accuracy, 83% sensitivity, and 75% specificity, while achieving an AUC of 87%. Models such as the Fuzzy ARTMAP applied to other datasets showed lower performance, with accuracy ranging between 60% and 80%, highlighting the variability in effectiveness across different methods.

**Table 1.** Performance of best classification models from the research studies.

Study	Model	Accuracy, %	Sensitivity, %	Specificity, %	AUC^a^, %	*F*_1_-score, %
Rahmat et al [28]	ANN^b^	—^c^	86.44	79.33	89.04	—
Collins et al [19]	ANN	80	80	—	—	—
Collins et al [19]	Fuzzy ARTMAP^d^	80	80	—	—	—
Nery et al [21]	JRIP^e^	80.10	85	81	82.60	75
Shenoy et al [29]	Random forest	—	87	—	91	86
Sonthayanon et al [25]	GA^f^	98.90	—	—	—	—
Nery Jr et al [22]	JRIP	—	84	99	—	—
Kulkarni et al [34]	U-Net^g^	98.02	—	—	—	—
Lopez et al [23]	J48^h^	70.5	—	—	—	—
Zhao et al [35]	Maxent^i^ model	—	—	—	96	—

a AUC: area under the curve.

b ANN: artificial neural network.

c Indicates metrics that were either not reported or not utilized in the original studies.

d ARTMAP: adaptive resonance theory mapping.

e JRIP: repeated incremental pruning to produce error reduction.

f GA: genetic algorithm.

g U-Net: U-Net convolutional neural network.

h J48: J48 decision tree.

i Maxent: maximum entropy.

**Table 2.** Performance of best prediction models from the research studies.

Study	Model	Accuracy, %	Sensitivity, %	Specificity, %	AUC^a^, %	MSE^b^	*R* ^2^	MAE^c^	RMSE^d^
Douchet et al [32]	SVR^e^	—^f^	—	—	—	0.19	—	—	—
Sonthayanon et al [24]	TAN^g^	—	—	—	89	—	—	—	—
Jayaramu et al [30]	RFC^h^	82.60	60	96.60	—	—	—	—	—
Galdino et al [10]	LASSO^i^	78.30	81.10	57.10	—	—	—	—	—
Ahangarcani et al [26]	SVM^j^	86.55	—	—	85.48	—	—	—	—
Mohammadinia et al [27]	GWR^k^	—	—	—	—	0.05	0.85	0.01	—
Douchet et al [33]	SVR	—	—	—	—	—	0.75	0.44	—
Thibeaux et al [31]	FFN^l^	—	—	—	—	—	—	—	0.67

a AUC: area under the curve.

b MSE: mean squared error.

c MAE: mean absolute error.

d RMSE: root mean squared error.

e SVR: support vector regression.

f Indicate metrics that were not reported or used in the original studies.

g TAN: tree augmented naïve network.

h RFC: random forest classifier.

i LASSO: least absolute shrinkage and selection operator regression.

j SVM: support vector machine.

k GWR: geographically weighted regression.

l FFN: feed forward network.

However, as with all model comparisons, it is not possible to directly compare the efficiency of models trained and evaluated on dissimilar datasets. To provide a meaningful evaluation, studies that implemented multiple machine learning methods on the same datasets were carefully selected for comparison. This allows for an accurate ranking of the algorithms based on their mean scores for accuracy, sensitivity, specificity, *F*_1_-score, and other metrics.

In several cases, regression models like SVR were assessed using MSE and *R*² values to gauge prediction performance. For instance, the SVR model in the Reunion Island dataset showed an MAE of 0.75 and an RMSE of 0.44, while other datasets revealed higher error rates, reflecting the challenges in prediction tasks [32].

The study also reveals that deep learning models, particularly those employing architectures like U-Net and CNN, achieved outstanding accuracy rates, with U-Net recording an accuracy of 98% [34]. Random forest models also performed consistently well, particularly in classification tasks, achieving high sensitivity and specificity.

**Figure 5.**
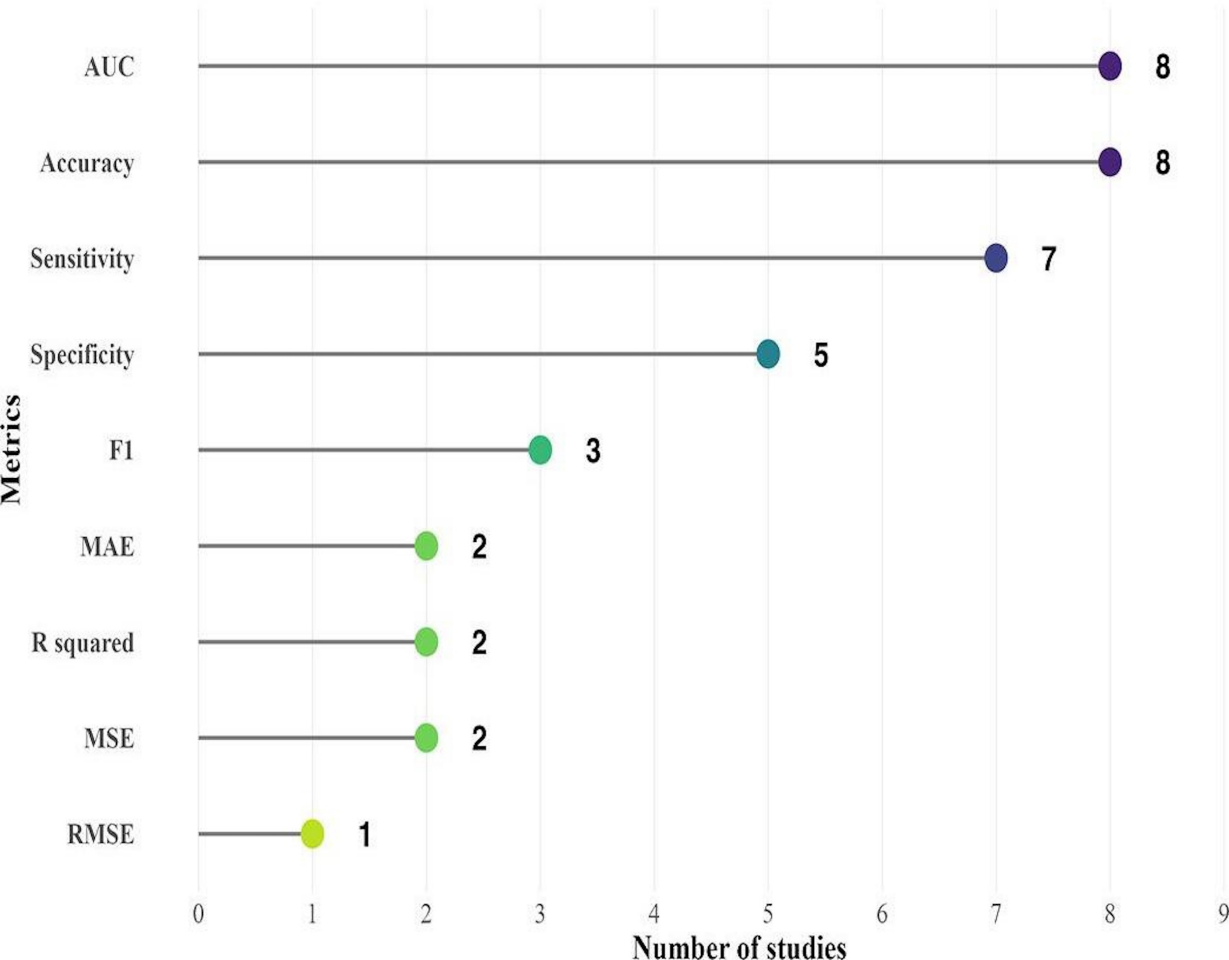
Number of algorithm performance metrics used in reviewed articles of dataset types used (public and private). AUC: area under the curve; F1: *F*_1_-score; MAE: mean absolute error; MSE: mean squared error; RMSE: root mean squared error.

Figure 5 shows the number of studies that reported various performance metrics, highlighting that accuracy and AUC were the most frequently used measures across the reviewed articles. This pattern reflects the widespread reliance on these metrics to evaluate classification performance in leptospirosis-related models. For instance, in one of the best-performing models, a genetic algorithm achieved an accuracy rate of 99%, significantly outperforming other models.

### Datasets and Data Sources

ML and DL studies for leptospirosis prediction and diagnosis rely on diverse datasets with distinct characteristics (see Table 3). Public datasets, typically sourced from government agencies, provide large-scale environmental and epidemiological data ideal for transmission modeling. For instance, the Thai Surveillance System [32] offered monthly rainfall measurements (0‐450 mm range), soil pH values (4.5‐8.2), and 30-meter resolution elevation data across 5 Southeast Asian countries from 2003 to 2018, comprising over 15,000 data points.

**Table 3.** Comprehensive dataset characteristics of included studies.

Study	Data type (source)	Data categories	Sample size	Temporal resolution	Spatial resolution
Douchet et al [32]	Public (Thai surveillance system)	Environmental: daily rainfall (mm)^a^, soil pH Climatic: max/min temperatures (°C) Topographic: elevation (SRTM^b^ 30m)	NS^c^	Monthly 2003‐2018	Regional (5 countries)
Rahmat et al [28]	Public (Malaysia Meteorological Department)	Meteorological: hourly rainfall (mm), RH^d^ (%) Clinical: PCR-confirmed^e^ cases	364 weeks	Weekly 2011‐2017	District-level (n=12)
Caicedo Torres et al [20]	Private (hospital records)	Clinical: fever days, liver enzymes (U/L) Demographic: age, gender, urban/rural	136 patients	Single admission	Hospital catchment
Nery et al [21]	Private (hospital records)	Clinical: serum creatinine (mg/dL) Epidemiological: rodent exposure index Geospatial: residence coordinates	4675 cases	2009‐2016	Household-level
Nery Jr et al [22]	Private (Gonçalo Moniz Institute [IGM], Federal University of Bahia [UFBA], Yale School of Public Health)	Clinical: patient records Epidemiological: risk factors, daily activities	4675 suspected cases (2046 confirmed, 2629 unconfirmed)	Retrospective (hospital and community cohort)	Hospital or community level (Salvador, Brazil)
Shenoy et al [29]	Private (medical records)	Clinical: jaundice severity scale (0‐3) Laboratory: ELISA^f^ optical densities Comorbidities: diabetes status	800 patients	Retrospective 5y	Single tertiary center
Sonthayanon et al [25]	Private (bacterial cultures)	Genomic: 16S rRNA^g^ sequences Proteomic: MALDI-TOF^h^ peaks (2k-20k m/z)	116 isolates	2015‐2018	Lab-level
Mayfield et al [24]	Private (serosurvey+ GIS^i^)	^j^Serological: MAT titers (1:50-1:6400) Environmental: livestock density/km² Village attributes: sanitation index	2152 people	Dry/wet season	GPS coordinates (82 villages)
Jayaramu et al [30]	Private (hydrological stations)	Streamflow (m³/s) Water level (m) Case reports (weekly)	517 weeks	Daily → weekly	Watershed-level
Galdino et al [10]	Private (hospital EMR^k^)	Vital signs: MAP (mmHg) Labs: creatinine (μmol/L) Outcomes: mortality	295 patients	2009‐2022	3 hospitals
Ahangarcani et al [26]	Mixed (MODIS^l^+ CDC^m^)	Satellite: NDVI^n^, LST (°C) Case reports: district-level Topography: slope (%)	1863 cases	Monthly 2009‐2014	District-level
Kulkarni et al [34]	Public (microscopy images)	Pixels: 256×256 RGB^o^ Annotations: spirochete masks	366 images	N/A	Pixel-level
Lopez et al [23]	Public (SINAN^p^ database)	Case reports: ICD-10^q^ coded Symptoms: 23-item checklist	890 cases	2007‐2016	State-level
Mohammadinia et al [27]	Public (National Ministry of Health and Treatment of Iran, National Centre of Statistics of Iran, Meteorology Agency of Iran)	Disease: positive ELISA test results	1186 positive cases (2009‐2011)	Longitudinal (2009‐2011)	District-level (Gilan Province, Iran)
Douchet et al [33]	Public (island surveillance)	Climate: CHIRPS^r^ rainfall (mm) Case counts: ministry reports	Monthly NS	2010‐2022	Island-level
Thibeaux et al [31]	Private (water monitoring)	Hydrological: turbidity (NTU)^s^ Microbiological: qPCR^t^ (copies/mL) Weather: 5-min rainfall	226 samples	Event-based	3 km² watershed
Zhao et al [35]	Public (China CDC)	Environmental: river density (km/km²) Socioeconomic: nightlight index Cases: lab-confirmed	2741 cases	Annual 2004‐2014	County-level

a Standard units of measurement (mm, °C, m, etc.).

b SRTM: Shuttle Radar Topography Mission.

c NS: not specified.

d RH: relative humidity .

e PCR: polymerase chain reaction.

f ELISA: Enzyme-Linked Immunosorbent Assay.

g rRNA: ribosomal ribonucleic acid.

h MALDI-TOF: Matrix-Assisted Laser Desorption/Ionization Time-of-Flight.

i GIS: Geographic Information System.

j MAT: microscopic agglutination test.

k EMR: electronic medical record.

l MODIS: Moderate Resolution Imaging Spectroradiometer.

m CDC: Centers for Disease Control and Prevention.

n NDVI: Normalized Difference Vegetation Index.

o RGB: red-green-blue color model.

p SINAN: Sistema de Informação de Agravos de Notificação.

q *ICD-10: International Classification of Diseases, Tenth Revision.*

r CHIRPS: Climate Hazards Group InfraRed Precipitation with Station data.

s NTU: Nephelometric Turbidity Unit.

t qPCR: quantitative polymerase chain reaction.

These datasets enabled regional risk prediction but lacked individual patient details. Similarly, Malaysia Meteorological Department records [28] provided 364 weeks of hourly rainfall data (0‐65mm/hr) and relative humidity (45%‐100%) paired with PCR-confirmed cases across 12 districts, demonstrating how high-resolution temporal data improves ANN-based outbreak forecasting.

Private clinical datasets, while smaller in scale, delivered granular patient-level information crucial for diagnostic accuracy. The Napoleón Franco Pareja Children’s Hospital dataset [20] included 136 pediatric cases with detailed clinical parameters: fever duration (1‐21 d), liver enzyme levels (AST 15‐980 U/L), and urban/rural residence markers. More extensive Brazilian hospital records [21] encompassed 4675 cases with serial creatinine measurements (0.2‐9.8 mg/dL) and household GPS coordinates, though missing 12% of lab results. These datasets typically included three key data categories: (1) clinical biomarkers (serum creatinine, MAT titers 1:50-1:6400), (2) demographic information (age, gender in 89% of studies), and (3) epidemiological risk factors (rodent exposure indices).

Advanced studies combined multiple data types to overcome individual limitations. Research in China [35] integrated 2741 CDC case reports with satellite-derived nighttime light indices (0‐63 DN values) and river density maps (0‐5.7 km/km²), achieving exceptional predictive performance (AUC 0.95‐0.96). Hydrological studies in New Caledonia [31] correlated 226 water samples (turbidity 0‐1,000 NTU, qPCR 10‐10⁶ copies/mL) with 5-minute rainfall events, demonstrating how microenvironmental data enhances transmission understanding. These multimodal approaches compensated for individual dataset constraints through: (1) temporal complementarity (monthly climate + daily case reports), (2) spatial layering (watershed hydrology+ village coordinates), and (3) clinical-environmental linkages (serum markers + livestock density).

## Discussion

### Principal Findings

This systematic review of 17 studies (2012‐2024) shows that while ML and DL techniques achieve promising accuracy (80%‐98%) in leptospirosis prediction and diagnosis, 3 critical limitations hinder clinical translation: (1) reliance on small, private clinical datasets limiting generalizability; (2) inconsistent validation methods, with only 11.8% employing temporal validation despite seasonality; and (3) underuse of advanced techniques like transfer learning (0% adoption) and data augmentation (5.9% adoption).

The most effective algorithms varied by task—SVR and Random Forest for prediction, versus U-Net CNNs for microscopy-based diagnosis—but all models faced challenges related to data quality, sample size, and geographic bias.

Textbox 1 shows the summary of the strengths and limitations identified in ML and DL studies for leptospirosis prediction and diagnosis.

Textbox 1. **Summary of strengths and limitations identified in machine learning (ML) and deep learning (DL) studies for leptospirosis prediction and diagnosis.**

Strengths:

- High predictive performance (80%‐98% accuracy).
- Variety of ML and DL algorithms applied (eg, random forests, support vector machines, and onvolutional neural networks).
- Integration of clinical and environmental data in some studies.
- Growing research interest and recent publications.

Limitations:

- Small, private datasets limit generalizability.
- Lack of external validation across datasets.
- Underuse of transfer learning and ensemble methods.
- Inconsistent evaluation metrics (accuracy, area under the curve, and sensitivity).

While these methods show strong performance (80%‐98% accuracy in some cases), their real-world applicability remains limited due to dataset constraints, validation inconsistencies, and underuse of advanced techniques such as transfer learning and ensemble learning. Addressing these gaps is essential to improve the robustness and clinical adoption of AI-driven leptospirosis diagnostics.

The most frequently used ML techniques for prediction tasks were SVR and Random Forest, while ANNs and CNNs, particularly U-Net, were commonly applied for diagnosis. This aligns with the increasing popularity of supervised ML methods in disease prediction.

Performance was primarily assessed using metrics such as accuracy, sensitivity, specificity, precision, and *F*_1_-score, although AUC offers a more comprehensive measure of model performance, especially in binary classification tasks. Hybrid methods often produced better outcomes, with Random Forest and U-Net demonstrating strong accuracy and sensitivity in leptospirosis classification tasks. For instance [29], achieved 87% accuracy and 91% sensitivity using Random Forest for diagnosis, suggesting that ensemble techniques could further enhance model performance.

Despite these advancements, there are still limitations in reaching clinician-level accuracy, especially when dealing with smaller datasets or limited training data. Acceptable performance thresholds varied, with some studies using an AUC score of 0.96 or higher as a benchmark, but this was not universally applied. In addition, most studies relied on private, hospital-based datasets, limiting generalizability. The lack of external validation raises concerns about potential bias, emphasizing the need for public datasets and standardized validation protocols to improve cross-study comparability.

Cross-validation was the predominant method for evaluating the models, with k-fold or leave-one-out cross-validation being most common. However, due to the relatively small sample sizes in many studies, the conclusions drawn may not be as robust. Split validation (eg, 80:20 or 70:30 splits) was also used, but inconsistencies across studies hindered direct model comparisons. Future work should prioritize establishing standardized protocols to enhance consistency and reliability in ML and DL evaluations.

One significant finding of this review was the absence of pretrained models. Most studies developed models from scratch, limiting the generalizability and scalability of these models. Transfer learning involves using a pretrained model as a starting point and fine-tuning it for a specific task. It has proven effective in various fields, particularly in image analysis and natural language processing, by significantly improving performance on tasks with limited data.

The lack of transfer learning in these studies suggests a potential area for future research, as it could enhance the performance of DL models in leptospirosis prediction and diagnosis, especially in cases where training data is limited. Similarly, data augmentation, which helps expand training datasets through transformations (eg, rotations, translations, or noise), was only used in 1 study, highlighting a missed opportunity to improve model robustness.

### Challenges and Research Gaps in ML and DL Applications for Leptospirosis Prediction and Diagnosis

While ML and DL have significant potential in leptospirosis research, this review also identified key findings that limit their broad application in clinical and public health settings.

#### Challenge 1: Limited Data Availability and Quality

A major challenge faced by the studies was the limited availability and quality of data. Many datasets, such as the one used in [21], had missing or incomplete data, which reduced the accuracy and generalizability of the models. Small sample sizes were a frequent issue as well, as observed in [20], where a dataset of only 136 patients resulted in high variability in model performance, especially for underrepresented cases like leptospirosis. Retrospective data collection posed further challenges, leading to biases in model training and evaluation, as noted by Shenoy et al [29].

#### Challenge 2: Generalizability and Regional Bias

Several studies, such as [28,33], identified biases in data collection, such as under- and over-reporting, regional disparities, and sampling biases, which hindered the broader applicability of the models. In addition, studies like [35] noted issues with the spatial resolution of environmental data, affecting the precision of fine-scale risk mapping. Moreover, the lack of external validation across different regions and datasets, as seen in studies like [10], increased the risk of overfitting and limited the broader applicability of findings.

The review showed inconsistent performance across different ML and DL algorithms. For example, models like SVM, ANN, and CNNs performed well, with accuracy reaching up to 98% [34]. However, advanced DL architectures like ResNet, Inception, and VGG were rarely used [25,33]. Similarly, none of the studies applied transfer learning, a technique that could enhance performance, particularly when data is scarce [10,28,36]. Beyond data related challenges, there are also limitations in the ML and DL techniques currently applied to leptospirosis diagnosis, as discussed next.

#### Challenge 3: Underuse of Advanced Techniques

Advanced ensemble techniques, such as XGBoost and Adaboost, were notably underused [26]. While Random Forest models and U-Net architectures performed well in specific tasks [29,34], ensemble methods could provide better predictive power when combining ML and DL approaches. The review also highlighted the minimal use of data augmentation techniques, which could help address the small sample size issues observed in many studies [34].

### Limitations of This Systematic Review

While this systematic review provides valuable insights, it has certain limitations. The heterogeneity of study designs, dataset sizes, and performance metrics precluded a meta-analysis, limiting our ability to provide a standardized comparison of model performances. In addition, the reliance on published studies may have introduced publication bias, as studies with less favorable results may have remained unpublished. Future systematic reviews should aim to standardize reporting metrics and ensure broader dataset accessibility to improve comparability across studies.

### Recommendations for Future Research

Based on these findings, future research in ML and DL applications for leptospirosis should focus on the following areas:

- Integration of advanced DL techniques: future studies should explore the potential of advanced DL architectures, such as ResNet and Inception, which are known to improve predictive performance, especially in image-based analysis.
- Leveraging pretrained models and transfer learning: research should investigate how pretrained models can be fine-tuned for leptospirosis applications, particularly in data-limited scenarios.
- Use of ensemble and hybrid approaches: advanced ensemble techniques like XGBoost and hybrid ML-DL approaches should be explored to improve model accuracy and robustness.
- Broader geographic representation: most studies focused on regions like Southeast Asia and Brazil, with limited research in other high-risk areas like Africa and Central America. Expanding research to these regions will improve model generalizability.

The primary goal moving forward is to aggregate a comprehensive dataset from diverse sources and develop a robust data library to enhance the accuracy and reliability of leptospirosis prediction models. Given the heterogeneity of data features across different studies—ranging from clinical records to environmental data—the focus will be on standardizing and harmonizing these features for better model integration. By consolidating larger and more varied datasets, we aim to improve model generalization and tackle current challenges related to small sample sizes and overfitting. This unified dataset will serve as a foundation for applying advanced techniques, such as transfer learning and ensemble methods, to further enhance the predictive power of ML and DL models in leptospirosis detection.

### Conclusion

This systematic review examined ML and DL techniques for leptospirosis prediction and diagnosis by analyzing algorithm performance, evaluation methods, and challenges. While models such as SVM, ANN, decision trees, and CNNs have shown strong predictive power, most studies have relied on private hospital-based datasets, limiting generalizability.

A key reason for the predominance of private datasets is that they often include detailed patient-level clinical information (eg, laboratory values, comorbidities, and symptoms) essential for developing diagnostic models. In contrast, available public datasets mainly provide aggregated epidemiological or environmental data, which, while valuable for outbreak prediction, lack the granular patient-specific features necessary for individual diagnosis. As a result, limited use of public datasets reflects the inherent constraints in the nature and detail of publicly available data, rather than a preference by researchers.

Furthermore, the lack of advanced techniques like transfer learning and ensemble methods remains a concern, along with small sample sizes and inconsistent validation protocols. Overall, while significant progress has been made, there is considerable potential to improve the accuracy and generalizability of leptospirosis prediction models by integrating more comprehensive datasets and adopting advanced AI methodologies in future research.

## Supplementary material

10.2196/67859 Multimedia Appendix 1 Search terms.

10.2196/67859 Multimedia Appendix 2 Agreement between human reviewers and ChatGPT-4o in study screening.

10.2196/67859 Multimedia Appendix 3 Quality assessment.

10.2196/67859 Multimedia Appendix 4 Characteristics of included studies.

10.2196/67859 Checklist 1 Preferred Reporting Items for Systematic Reviews and Meta-Analyses (PRISMA) checklist, detailing the sections, topics, checklist items, and their corresponding locations within the review.

## Abbreviation

AI: artificial intelligence
ANN: artificial neural network
AUC: area under the curve
CHARMS: Critical Appraisal and Data Extraction for Systematic Reviews of Prediction Modelling Studies
CNN: convolutional neural network
DL: deep learning
DT: decision tree
KNN: k-nearest neighbor
LLM: large language model
MAE: mean absolute error
MAT: microscopic agglutination test
ML: machine learning
MLP: multilayer perceptron
MSE: mean squared error
PCR: polymerase chain reaction
PRISMA: Preferred Reporting Items for Systematic Reviews and Meta-Analyses
PROBAST: Prediction model Risk of Bias Assessment Tool
RDT: rapid diagnostic test
RMSE: root mean squared error
SVM: support vector machine
SVR: support vector regression
TAN: tree augmented naive
