# Supplementary material for: Machine Learning and Deep Learning Techniques for Prediction and Diagnosis of Leptospirosis: Systematic Literature Review
Source: JMIR Med Inform. 2025 May 29;13:e67859. doi: 10.2196/67859 (PMC12140502; doi:10.2196/67859)
Supplement: Multimedia Appendix 1 [file medinform-v13-e67859-s001.docx]

**APPENDIX 1: SEARCH TERMS**

**Web of Science**

 (TI=(leptospirosis) OR AB=(leptospirosis)) AND (TI=("deep learning" OR "machine learning") OR AB=("deep learning" OR "machine learning"))

**PubMed**

(leptospirosis AND ("deep learning" OR "machine learning"))

**Scopus**

(TITLE-ABS-KEY(leptospirosis) AND TITLE-ABS-KEY("deep learning" OR "machine learning"))

**IEEE**

("Document Title":leptospirosis OR "Abstract":leptospirosis) AND ("Document Title":"deep learning" OR "Document Title":"machine learning" OR "Abstract":"deep learning" OR "Abstract":"machine learning")

**Google Scholar**

leptospirosis AND ("deep learning" OR "machine learning")
