# Supplementary material for: Machine Learning and Deep Learning Techniques for Prediction and Diagnosis of Leptospirosis: Systematic Literature Review
Source: JMIR Med Inform. 2025 May 29;13:e67859. doi: 10.2196/67859 (PMC12140502; doi:10.2196/67859)
Supplement: Multimedia Appendix 2 [file medinform-v13-e67859-s002.docx]

Appendix 2. Agreement Between Human Reviewers and ChatGPT-4o in Study Screening

| Article ID | Year | ChatGPT-4o Decision | B.R. Decision | A.J. Decision | S.S. Decision | % Agreement | Final Decision | Assessment | Final Decision |
| --- | --- | --- | --- | --- | --- | --- | --- | --- | --- |
| LEP-001 | 2022 | Include | Include | Include | Include | 100% | Include | Included in final 17 studies | Include |
| LEP-002 | 2020 | Include | Include | Include | Include | 100% | Include | Included in final 17 studies | Include |
| LEP-003 | 2012 | Exclude | Exclude | Exclude | Exclude | 100% | Include | Included in final 17 studies | Include |
| LEP-004 | 2019 | Exclude | Exclude | Exclude | Exclude | 100% | Exclude | Excluded | Exclude |
| LEP-005 | 2021 | Exclude | Exclude | Exclude | Exclude | 100% | Exclude | Excluded | Exclude |
| LEP-006 | 2017 | Exclude | Exclude | Exclude | Exclude | 100% | Exclude | Excluded | Exclude |
| LEP-007 | 2016 | Exclude | Exclude | Exclude | Exclude | 100% | Exclude | Excluded | Exclude |
| LEP-008 | 2021 | Exclude | Exclude | Exclude | Exclude | 100% | Exclude | Excluded | Exclude |
| LEP-009 | 2017 | Exclude | Exclude | Exclude | Include | 75% | Exclude | Excluded | Exclude |
| LEP-010 | 2017 | Exclude | Exclude | Exclude | Exclude | 100% | Exclude | Excluded | Exclude |
| LEP-011 | 2023 | Exclude | Exclude | Exclude | Exclude | 100% | Exclude | Excluded | Exclude |
| LEP-012 | 2019 | Exclude | Exclude | Exclude | Exclude | 100% | Exclude | Excluded | Exclude |
| LEP-013 | 2024 | Exclude | Exclude | Exclude | Exclude | 100% | Exclude | Excluded | Exclude |
| LEP-014 | 2023 | Exclude | Exclude | Exclude | Exclude | 100% | Exclude | Excluded | Exclude |
| LEP-015 | 2021 | Exclude | Include | Exclude | Exclude | 75% | Exclude | Excluded | Exclude |
| LEP-016 | 2017 | Exclude | Exclude | Exclude | Exclude | 100% | Exclude | Excluded | Exclude |
| LEP-017 | 2016 | Exclude | Exclude | Exclude | Exclude | 100% | Exclude | Excluded | Exclude |
| LEP-018 | 2024 | Exclude | Exclude | Exclude | Exclude | 100% | Exclude | Excluded | Exclude |
| LEP-019 | 2022 | Exclude | Exclude | Exclude | Include | 75% | Exclude | Excluded | Exclude |
| LEP-020 | 2017 | Exclude | Include | Exclude | Exclude | 75% | Exclude | Excluded | Exclude |
| LEP-021 | 2017 | Exclude | Exclude | Exclude | Exclude | 100% | Exclude | Excluded | Exclude |
| LEP-022 | 2016 | Include | Exclude | Exclude | Exclude | 100% | Include | Included in final 17 studies | Include |
| LEP-023 | 2019 | Exclude | Exclude | Exclude | Exclude | 100% | Exclude | Excluded | Exclude |
| LEP-024 | 2022 | Include | Exclude | Exclude | Exclude | 100% | Include | Included in final 17 studies | Include |
| LEP-025 | 2023 | Exclude | Exclude | Exclude | Exclude | 100% | Exclude | Excluded | Exclude |
| LEP-026 | 2019 | Include | Include | Include | Include | 100 | Include | Included in final 17 studies | Include |
| LEP-027 | 2017 | Include | Include | Exclude | Exclude | 75% | Include | Included in final 17 studies | Include |
| LEP-028 | 2019 | Exclude | Exclude | Exclude | Exclude | 100% | Exclude | Excluded | Exclude |
| LEP-029 | 2018 | Include | Include | Include | Include | 100% | Include | Included in final 17 studies | Include |
| LEP-030 | 2023 | Include | Include | Include | Include | 100% | Include | Included in final 17 studies | Include |
| LEP-031 | 2024 | Exclude | Exclude | Exclude | Exclude | 100% | Exclude | Excluded | Exclude |
| LEP-032 | 2023 | Exclude | Exclude | Exclude | Exclude | 100% | Exclude | Excluded | Exclude |
| LEP-033 | 2019 | Exclude | Exclude | Exclude | Exclude | 100% | Exclude | Excluded | Exclude |
| LEP-034 | 2016 | Exclude | Exclude | Exclude | Exclude | 100% | Exclude | Excluded | Exclude |
| LEP-035 | 2023 | Include | Include | Include | Include | 100% | Include | Included in final 17 studies | Include |
| LEP-036 | 2016 | Include | Include | Include | Include | 100% | Include | Included in final 17 studies | Include |
| LEP-037 | 2012 | Exclude | Exclude | Exclude | Exclude | 100% | Exclude | Excluded | Exclude |
| LEP-038 | 2019 | Include | Exclude | Exclude | Exclude | 75% | Include | Included in final 17 studies | Include |
| LEP-039 | 2022 | Exclude | Exclude | Exclude | Exclude | 100% | Exclude | Excluded | Exclude |
| LEP-040 | 2020 | Include | Exclude | Exclude | Exclude | 100% | Include | Included in final 17 studies | Include |
| LEP-041 | 2022 | Include | Include | Include | Include | 100% | Include | Included in final 17 studies | Include |
| LEP-042 | 2018 | Exclude | Exclude | Exclude | Exclude | 100% | Exclude | Excluded | Exclude |
| LEP-043 | 2023 | Exclude | Exclude | Exclude | Exclude | 100% | Exclude | Excluded | Exclude |
| LEP-044 | 2023 | Exclude | Exclude | Exclude | Exclude | 100% | Exclude | Excluded | Exclude |
| LEP-045 | 2019 | Exclude | Exclude | Exclude | Exclude | 100% | Exclude | Excluded | Exclude |
| LEP-046 | 2017 | Include | Exclude | Exclude | Exclude | 75% | Include | Included in final 17 studies | Include |
| LEP-047 | 2020 | Exclude | Exclude | Exclude | Exclude | 100% | Exclude | Excluded | Exclude |
| LEP-048 | 2022 | Exclude | Exclude | Exclude | Exclude | 100% | Exclude | Excluded | Exclude |
| LEP-049 | 2017 | Exclude | Exclude | Exclude | Exclude | 100% | Exclude | Excluded | Exclude |
| LEP-050 | 2024 | Exclude | Exclude | Exclude | Exclude | 100% | Exclude | Excluded | Exclude |
| LEP-051 | 2024 | Exclude | Exclude | Exclude | Exclude | 100% | Exclude | Excluded | Exclude |
| LEP-052 | 2024 | Exclude | Exclude | Exclude | Exclude | 100% | Exclude | Excluded | Exclude |
| LEP-053 | 2016 | Exclude | Exclude | Exclude | Exclude | 100% | Exclude | Excluded | Exclude |
| LEP-054 | 2024 | Exclude | Exclude | Exclude | Exclude | 100% | Exclude | Excluded | Exclude |
| LEP-055 | 2024 | Include | Exclude | Exclude | Exclude | 75% | Include | Included in final 17 studies | Include |
| LEP-056 | 2023 | Exclude | Exclude | Exclude | Exclude | 100% | Exclude | Excluded | Exclude |
| LEP-057 | 2016 | Exclude | Exclude | Exclude | Exclude | 100% | Exclude | Excluded | Exclude |
| LEP-058 | 2024 | Include | Exclude | Exclude | Exclude | 50 | Include | Included in final 17 studies | Include |
| LEP-059 | 2017 | Include | Exclude | Exclude | Exclude | 75% | Exclude | Excluded | Exclude |
| LEP-060 | 2018 | Exclude | Exclude | Exclude | Exclude | 100% | Exclude | Excluded | Exclude |
| LEP-061 | 2023 | Include | Exclude | Include | Exclude | 50% | Exclude | Excluded | Exclude |
| LEP-062 | 2023 | Include | Exclude | Exclude | Exclude | 75% | Exclude | Excluded | Exclude |
| LEP-063 | 2019 | Exclude | Exclude | Exclude | Exclude | 100% | Exclude | Excluded | Exclude |
| LEP-064 | 2019 | Exclude | Exclude | Exclude | Exclude | 100% | Exclude | Excluded | Exclude |
| LEP-065 | 2020 | Include | Exclude | Exclude | Exclude | 100% | Exclude | Excluded | Exclude |
| LEP-066 | 2022 | Include | Exclude | Exclude | Exclude | 75% | Exclude | Excluded | Exclude |
| LEP-067 | 2017 | Exclude | Exclude | Exclude | Exclude | 100% | Exclude | Excluded | Exclude |
| LEP-068 | 2024 | Exclude | Exclude | Exclude | Exclude | 100% | Exclude | Excluded | Exclude |
| LEP-069 | 2024 | Exclude | Exclude | Exclude | Exclude | 100% | Exclude | Excluded | Exclude |
| LEP-070 | 2024 | Exclude | Exclude | Exclude | Exclude | 100% | Exclude | Excluded | Exclude |
| LEP-071 | 2016 | Exclude | Exclude | Exclude | Exclude | 100% | Exclude | Excluded | Exclude |
| LEP-072 | 2024 | Exclude | Exclude | Exclude | Exclude | 100% | Exclude | Excluded | Exclude |
| LEP-073 | 2023 | Include | Exclude | Exclude | Exclude | 100% | Exclude | Excluded | Exclude |
| LEP-074 | 2023 | Include | Exclude | Exclude | Exclude | 75% | Exclude | Excluded | Exclude |
| LEP-075 | 2016 | Exclude | Exclude | Exclude | Exclude | 100% | Exclude | Excluded | Exclude |
| LEP-076 | 2020 | Exclude | Exclude | Exclude | Exclude | 100% | Exclude | Excluded | Exclude |
| LEP-077 | 2023 | Exclude | Exclude | Exclude | Exclude | 100% | Exclude | Excluded | Exclude |
| LEP-078 | 2019 | Include | Include | Include | Include | 100% | Exclude | Excluded | Exclude |
| LEP-079 | 2021 | Include | Exclude | Exclude | Exclude | 100% | Exclude | Excluded | Exclude |
| LEP-080 | 2020 | Exclude | Exclude | Exclude | Exclude | 100% | Exclude | Excluded | Exclude |
| LEP-081 | 2023 | Exclude | Exclude | Exclude | Exclude | 100% | Exclude | Excluded | Exclude |
| LEP-082 | 2019 | Include | Include | Include | Include | 100% | Exclude | Excluded | Exclude |
| LEP-083 | 2021 | Include | Include | Include | Include | 100% | Exclude | Excluded | Exclude |
| LEP-084 | 2017 | Exclude | Exclude | Exclude | Exclude | 100% | Exclude | Excluded | Exclude |
| LEP-085 | 2016 | Exclude | Exclude | Exclude | Exclude | 100% | Exclude | Excluded | Exclude |
| LEP-086 | 2020 | Exclude | Exclude | Exclude | Exclude | 100% | Exclude | Excluded | Exclude |
| LEP-087 | 2023 | Exclude | Exclude | Exclude | Exclude | 100% | Exclude | Excluded | Exclude |
| LEP-088 | 2019 | Include | Include | Include | Include | 100% | Exclude | Excluded | Exclude |
| LEP-089 | 2021 | Exclude | Exclude | Exclude | Exclude | 100% | Exclude | Excluded | Exclude |
| LEP-090 | 2017 | Exclude | Exclude | Exclude | Exclude | 100% | Exclude | Excluded | Exclude |
| LEP-091 | 2016 | Exclude | Exclude | Exclude | Exclude | 100% | Exclude | Excluded | Exclude |
| LEP-092 | 2021 | Include | Exclude | Exclude | Exclude | 100% | Exclude | Excluded | Exclude |
| LEP-093 | 2017 | Include | Include | Include | Include | 100% | Exclude | Excluded | Exclude |
| LEP-094 | 2017 | Include | Include | Include | Include | 100% | Exclude | Excluded | Exclude |
| LEP-095 | 2023 | Include | Include | Include | Include | 100% | Exclude | Excluded | Exclude |
| LEP-096 | 2019 | Include | Include | Include | Include | 100% | Exclude | Excluded | Exclude |
| LEP-097 | 2024 | Exclude | Exclude | Exclude | Exclude | 100% | Exclude | Excluded | Exclude |
| LEP-098 | 2023 | Exclude | Exclude | Exclude | Exclude | 100% | Exclude | Excluded | Exclude |
| LEP-099 | 2023 | Exclude | Exclude | Exclude | Exclude | 100% | Exclude | Excluded | Exclude |
| LEP-100 | 2023 | Exclude | Exclude | Exclude | Exclude | 100% | Exclude | Excluded | Exclude |
| LEP-101 | 2016 | Exclude | Exclude | Exclude | Exclude | 100% | Exclude | Excluded | Exclude |
| LEP-102 | 2019 | Exclude | Exclude | Exclude | Exclude | 100% | Exclude | Excluded | Exclude |
| LEP-103 | 2017 | Exclude | Exclude | Exclude | Exclude | 100% | Exclude | Excluded | Exclude |
| LEP-104 | 2023 | Exclude | Exclude | Exclude | Exclude | 100% | Exclude | Excluded | Exclude |
| LEP-105 | 2023 | Exclude | Exclude | Exclude | Exclude | 100% | Exclude | Excluded | Exclude |
| LEP-106 | 2016 | Exclude | Exclude | Exclude | Exclude | 100% | Exclude | Excluded | Exclude |
| LEP-107 | 2019 | Include | Include | Include | Include | 100% | Exclude | Excluded | Exclude |
| LEP-108 | 2023 | Exclude | Exclude | Exclude | Exclude | 100% | Exclude | Excluded | Exclude |
| LEP-109 | 2023 | Include | Include | Include | Include | 100% | Exclude | Excluded | Exclude |
| LEP-110 | 2016 | Include | Include | Include | Include | 100% | Exclude | Excluded | Exclude |
| LEP-111 | 2019 | Exclude | Exclude | Exclude | Exclude | 100% | Exclude | Excluded | Exclude |
| LEP-112 | 2023 | Include | Exclude | Exclude | Exclude | 75% | Exclude | Excluded | Exclude |
| LEP-113 | 2023 | Exclude | Exclude | Exclude | Exclude | 100% | Exclude | Excluded | Exclude |
| LEP-114 | 2016 | Include | Exclude | Exclude | Exclude | 75% | Exclude | Excluded | Exclude |
| LEP-115 | 2019 | Include | Include | Include | Include | 100% | Exclude | Excluded | Exclude |
| LEP-116 | 2017 | Exclude | Exclude | Exclude | Exclude | 100% | Exclude | Excluded | Exclude |
| LEP-117 | 2018 | Exclude | Exclude | Exclude | Exclude | 100% | Exclude | Excluded | Exclude |
| LEP-118 | 2023 | Exclude | Exclude | Exclude | Exclude | 100% | Exclude | Excluded | Exclude |
| LEP-119 | 2023 | Exclude | Exclude | Exclude | Exclude | 100% | Exclude | Excluded | Exclude |
| LEP-120 | 2023 | Exclude | Exclude | Exclude | Exclude | 100% | Exclude | Excluded | Exclude |
| LEP-121 | 2023 | Exclude | Exclude | Exclude | Exclude | 100% | Exclude | Excluded | Exclude |
| LEP-122 | 2016 | Include | Exclude | Exclude | Exclude | 75% | Exclude | Excluded | Exclude |
| LEP-123 | 2019 | Include | Exclude | Exclude | Exclude | 75% | Exclude | Excluded | Exclude |
| LEP-124 | 2017 | Exclude | Exclude | Exclude | Exclude | 100% | Exclude | Excluded | Exclude |
| LEP-125 | 2018 | Exclude | Exclude | Exclude | Exclude | 100% | Exclude | Excluded | Exclude |
| LEP-126 | 2023 | Include | Exclude | Exclude | Exclude | 75% | Exclude | Excluded | Exclude |
| LEP-127 | 2023 | Exclude | Exclude | Exclude | Exclude | 100% | Exclude | Excluded | Exclude |
| LEP-128 | 2019 | Exclude | Exclude | Exclude | Exclude | 100% | Exclude | Excluded | Exclude |
| LEP-129 | 2023 | Exclude | Exclude | Exclude | Exclude | 100% | Exclude | Excluded | Exclude |
| LEP-130 | 2023 | Exclude | Exclude | Exclude | Exclude | 100% | Exclude | Excluded | Exclude |
| LEP-131 | 2016 | Exclude | Exclude | Exclude | Exclude | 100% | Exclude | Excluded | Exclude |
| LEP-132 | 2019 | Include | Exclude | Exclude | Exclude | 75% | Exclude | Excluded | Exclude |
| LEP-133 | 2017 | Exclude | Exclude | Exclude | Exclude | 100% | Exclude | Excluded | Exclude |
| LEP-134 | 2018 | Include | Exclude | Exclude | Exclude | 75% | Exclude | Excluded | Exclude |
| LEP-135 | 2023 | Exclude | Exclude | Exclude | Exclude | 100% | Exclude | Excluded | Exclude |
| LEP-136 | 2023 | Exclude | Exclude | Exclude | Exclude | 100% | Exclude | Excluded | Exclude |
| LEP-137 | 2019 | Exclude | Exclude | Exclude | Exclude | 100% | Exclude | Excluded | Exclude |
| LEP-138 | 2023 | Include | Exclude | Exclude | Exclude | 75% | Exclude | Excluded | Exclude |
| LEP-139 | 2023 | Exclude | Exclude | Exclude | Exclude | 100% | Exclude | Excluded | Exclude |
| LEP-140 | 2016 | Exclude | Exclude | Exclude | Exclude | 100% | Exclude | Excluded | Exclude |
| LEP-141 | 2019 | Include | Exclude | Exclude | Exclude | 75% | Exclude | Excluded | Exclude |
| LEP-142 | 2017 | Exclude | Exclude | Exclude | Exclude | 100% | Exclude | Excluded | Exclude |
| LEP-143 | 2018 | Exclude | Exclude | Exclude | Exclude | 100% | Exclude | Excluded | Exclude |
| LEP-144 | 2023 | Exclude | Exclude | Exclude | Exclude | 100% | Exclude | Excluded | Exclude |
| LEP-145 | 2023 | Include | Exclude | Exclude | Exclude | 75% | Exclude | Excluded | Exclude |
| LEP-146 | 2019 | Exclude | Exclude | Exclude | Exclude | 100% | Exclude | Excluded | Exclude |
| LEP-147 | 2023 | Exclude | Exclude | Exclude | Exclude | 100% | Exclude | Excluded | Exclude |
| LEP-148 | 2023 | Exclude | Exclude | Exclude | Exclude | 100% | Exclude | Excluded | Exclude |
| LEP-149 | 2016 | Exclude | Exclude | Exclude | Exclude | 100% | Exclude | Excluded | Exclude |
| LEP-150 | 2019 | Exclude | Exclude | Exclude | Exclude | 100% | Exclude | Excluded | Exclude |
| LEP-151 | 2017 | Include | Exclude | Exclude | Exclude | 75% | Exclude | Excluded | Exclude |
| LEP-152 | 2018 | Exclude | Exclude | Exclude | Exclude | 100% | Exclude | Excluded | Exclude |
| LEP-153 | 2023 | Include | Include | Include | Include | 100% | Exclude | Excluded | Exclude |
| LEP-154 | 2023 | Include | Include | Include | Include | 100% | Exclude | Excluded | Exclude |
| LEP-155 | 2019 | Exclude | Exclude | Exclude | Exclude | 100% | Exclude | Excluded | Exclude |
| LEP-156 | 2019 | Include | Exclude | Exclude | Exclude | 75% | Exclude | Excluded | Exclude |
| LEP-157 | 2023 | Exclude | Exclude | Exclude | Exclude | 100% | Exclude | Excluded | Exclude |
| LEP-158 | 2023 | Exclude | Exclude | Exclude | Exclude | 100% | Exclude | Excluded | Exclude |
| LEP-159 | 2016 | Include | Include | Include | Include | 100% | Exclude | Excluded | Exclude |
| LEP-160 | 2019 | Exclude | Exclude | Exclude | Exclude | 100% | Exclude | Excluded | Exclude |
| LEP-161 | 2017 | Exclude | Exclude | Exclude | Exclude | 100% | Exclude | Excluded | Exclude |
| LEP-162 | 2018 | Include | Exclude | Exclude | Exclude | 75% | Exclude | Excluded | Exclude |
| LEP-163 | 2023 | Exclude | Exclude | Exclude | Exclude | 100% | Exclude | Excluded | Exclude |
| LEP-164 | 2023 | Exclude | Exclude | Exclude | Exclude | 100% | Exclude | Excluded | Exclude |
| LEP-165 | 2019 | Include | Exclude | Exclude | Exclude | 75% | Exclude | Excluded | Exclude |
| LEP-166 | 2019 | Exclude | Exclude | Exclude | Exclude | 100% | Exclude | Excluded | Exclude |
| LEP-167 | 2020 | Include | Exclude | Exclude | Exclude | 75% | Exclude | Excluded | Exclude |
| LEP-168 | 2022 | Exclude | Exclude | Exclude | Exclude | 100% | Exclude | Excluded | Exclude |
| LEP-169 | 2024 | Exclude | Exclude | Exclude | Exclude | 100% | Exclude | Excluded | Exclude |
| LEP-170 | 2021 | Exclude | Exclude | Exclude | Exclude | 100% | Exclude | Excluded | Exclude |
| LEP-171 | 2023 | Include | Exclude | Exclude | Exclude | 75% | Exclude | Excluded | Exclude |
| LEP-172 | 2023 | Exclude | Exclude | Exclude | Exclude | 100% | Exclude | Excluded | Exclude |
| LEP-173 | 2016 | Exclude | Exclude | Exclude | Exclude | 100% | Exclude | Excluded | Exclude |
| LEP-174 | 2019 | Exclude | Exclude | Exclude | Exclude | 100% | Exclude | Excluded | Exclude |
| LEP-175 | 2017 | Exclude | Exclude | Exclude | Exclude | 100% | Exclude | Excluded | Exclude |
| LEP-176 | 2018 | Include | Exclude | Exclude | Exclude | 75% | Exclude | Excluded | Exclude |
| LEP-177 | 2023 | Exclude | Exclude | Exclude | Exclude | 100% | Exclude | Excluded | Exclude |
| LEP-178 | 2023 | Exclude | Exclude | Exclude | Exclude | 100% | Exclude | Excluded | Exclude |
| LEP-179 | 2019 | Exclude | Exclude | Exclude | Exclude | 100% | Exclude | Excluded | Exclude |
| LEP-180 | 2023 | Exclude | Exclude | Exclude | Exclude | 100% | Exclude | Excluded | Exclude |
| LEP-181 | 2023 | Include | Exclude | Exclude | Exclude | 75% | Exclude | Excluded | Exclude |
| LEP-182 | 2016 | Exclude | Exclude | Exclude | Exclude | 100% | Exclude | Excluded | Exclude |
| LEP-183 | 2019 | Exclude | Exclude | Exclude | Exclude | 100% | Exclude | Excluded | Exclude |
| LEP-184 | 2017 | Include | Exclude | Exclude | Exclude | 75% | Exclude | Excluded | Exclude |
| LEP-185 | 2018 | Exclude | Exclude | Exclude | Exclude | 100% | Exclude | Excluded | Exclude |
| LEP-186 | 2023 | Exclude | Exclude | Exclude | Exclude | 100% | Exclude | Excluded | Exclude |
| LEP-187 | 2023 | Include | Include | Include | Include | 100% | Exclude | Excluded | Exclude |
| LEP-188 | 2019 | Exclude | Exclude | Exclude | Exclude | 100% | Exclude | Excluded | Exclude |
| LEP-189 | 2019 | Exclude | Exclude | Exclude | Exclude | 100% | Exclude | Excluded | Exclude |
| LEP-190 | 2023 | Include | Exclude | Exclude | Exclude | 75% | Exclude | Excluded | Exclude |
| LEP-191 | 2016 | Exclude | Exclude | Exclude | Exclude | 100% | Exclude | Excluded | Exclude |
| LEP-192 | 2019 | Exclude | Exclude | Exclude | Exclude | 100% | Exclude | Excluded | Exclude |
| LEP-193 | 2017 | Include | Exclude | Exclude | Exclude | 75% | Exclude | Excluded | Exclude |
| LEP-194 | 2018 | Include | Include | Include | Include | 100% | Exclude | Excluded | Exclude |
| LEP-195 | 2023 | Include | Include | Include | Include | 100% | Exclude | Excluded | Exclude |
| LEP-196 | 2023 | Exclude | Exclude | Exclude | Exclude | 100% | Exclude | Excluded | Exclude |
| LEP-197 | 2016 | Exclude | Exclude | Exclude | Exclude | 100% | Exclude | Excluded | Exclude |
| LEP-198 | 2019 | Include | Exclude | Exclude | Exclude | 75% | Exclude | Excluded | Exclude |
| LEP-199 | 2017 | Exclude | Exclude | Exclude | Exclude | 100% | Exclude | Excluded | Exclude |
| LEP-200 | 2018 | Include | Include | Include | Include | 100% | Exclude | Excluded | Exclude |
| LEP-201 | 2023 | Include | Include | Include | Include | 100% | Exclude | Excluded | Exclude |
| LEP-202 | 2016 | Include | Exclude | Exclude | Exclude | 75% | Exclude | Excluded | Exclude |
| LEP-203 | 2019 | Exclude | Exclude | Exclude | Exclude | 100% | Exclude | Excluded | Exclude |
| LEP-204 | 2017 | Exclude | Exclude | Exclude | Exclude | 100% | Exclude | Excluded | Exclude |
| LEP-205 | 2018 | Include | Exclude | Include | Exclude | 50% | Exclude | Excluded | Exclude |
| LEP-206 | 2023 | Exclude | Exclude | Exclude | Exclude | 100% | Exclude | Excluded | Exclude |
| LEP-207 | 2023 | Include | Include | Include | Include | 100% | Exclude | Excluded | Exclude |
| LEP-208 | 2019 | Include | Include | Include | Include | 100% | Exclude | Excluded | Exclude |
| LEP-209 | 2016 | Include | Include | Include | Include | 100% | Exclude | Excluded | Exclude |
| LEP-210 | 2019 | Include | Include | Include | Include | 100% | Exclude | Excluded | Exclude |
| LEP-211 | 2023 | Include | Exclude | Exclude | Exclude | 75% | Exclude | Excluded | Exclude |
| LEP-212 | 2023 | Include | Include | Include | Include | 100% | Exclude | Excluded | Exclude |
| LEP-213 | 2016 | Include | Include | Include | Include | 100% | Exclude | Excluded | Exclude |
| LEP-214 | 2019 | Include | Include | Include | Include | 100% | Exclude | Excluded | Exclude |
| LEP-215 | 2023 | Include | Include | Include | Include | 100% | Exclude | Excluded | Exclude |
| LEP-216 | 2023 | Include | Include | Include | Include | 100% | Exclude | Excluded | Exclude |
| LEP-217 | 2016 | Include | Include | Include | Include | 100% | Exclude | Excluded | Exclude |
| LEP-218 | 2019 | Include | Include | Include | Include | 100% | Exclude | Excluded | Exclude |
| LEP-219 | 2017 | Include | Include | Include | Include | 100% | Exclude | Excluded | Exclude |
| LEP-220 | 2016 | Include | Include | Include | Include | 100% | Exclude | Excluded | Exclude |
| LEP-221 | 2019 | Include | Include | Include | Include | 100% | Exclude | Excluded | Exclude |
| LEP-222 | 2023 | Include | Include | Include | Include | 100% | Exclude | Excluded | Exclude |
| LEP-223 | 2023 | Include | Include | Include | Include | 100% | Exclude | Excluded | Exclude |
| LEP-224 | 2016 | Include | Include | Include | Include | 100% | Exclude | Excluded | Exclude |
| LEP-225 | 2019 | Include | Include | Include | Include | 100% | Exclude | Excluded | Exclude |
| LEP-226 | 2023 | Include | Include | Include | Include | 100% | Exclude | Excluded | Exclude |
| LEP-227 | 2023 | Include | Include | Include | Include | 100% | Exclude | Excluded | Exclude |
| LEP-228 | 2016 | Include | Include | Include | Include | 100% | Exclude | Excluded | Exclude |
| LEP-229 | 2019 | Include | Include | Include | Include | 100% | Exclude | Excluded | Exclude |
| LEP-230 | 2017 | Exclude | Exclude | Exclude | Exclude | 100% | Exclude | Excluded | Exclude |
| LEP-231 | 2016 | Include | Include | Include | Include | 100% | Exclude | Excluded | Exclude |
| LEP-232 | 2019 | Include | Include | Include | Include | 100% | Exclude | Excluded | Exclude |
| LEP-233 | 2023 | Include | Include | Include | Include | 100% | Exclude | Excluded | Exclude |
| LEP-234 | 2023 | Exclude | Exclude | Exclude | Exclude | 100% | Exclude | Excluded | Exclude |
| LEP-235 | 2016 | Include | Exclude | Include | Exclude | 50% | Exclude | Excluded | Exclude |
| LEP-236 | 2019 | Exclude | Exclude | Exclude | Exclude | 100% | Exclude | Excluded | Exclude |
| LEP-237 | 2023 | Exclude | Exclude | Exclude | Exclude | 100% | Exclude | Excluded | Exclude |
| LEP-238 | 2023 | Exclude | Exclude | Exclude | Exclude | 100% | Exclude | Excluded | Exclude |
| LEP-239 | 2016 | Include | Exclude | Exclude | Exclude | 75% | Exclude | Excluded | Exclude |
| LEP-240 | 2016 | Exclude | Exclude | Exclude | Exclude | 100% | Exclude | Excluded | Exclude |
| LEP-241 | 2019 | Include | Exclude | Exclude | Exclude | 75% | Exclude | Excluded | Exclude |
| LEP-242 | 2023 | Exclude | Exclude | Exclude | Exclude | 100% | Exclude | Excluded | Exclude |
| LEP-243 | 2023 | Include | Exclude | Include | Exclude | 50% | Exclude | Excluded | Exclude |
| LEP-244 | 2016 | Exclude | Exclude | Exclude | Exclude | 100% | Exclude | Excluded | Exclude |
| LEP-245 | 2019 | Exclude | Exclude | Exclude | Exclude | 100% | Exclude | Excluded | Exclude |
| LEP-246 | 2023 | Exclude | Exclude | Exclude | Exclude | 100% | Exclude | Excluded | Exclude |
| LEP-247 | 2023 | Include | Include | Include | Include | 100% | Exclude | Excluded | Exclude |
| LEP-248 | 2016 | Include | Include | Include | Include | 100% | Exclude | Excluded | Exclude |
| LEP-249 | 2019 | Include | Include | Include | Include | 100% | Exclude | Excluded | Exclude |
| LEP-250 | 2017 | Include | Include | Include | Include | 100% | Exclude | Excluded | Exclude |
| LEP-251 | 2018 | Exclude | Exclude | Exclude | Exclude | 100% | Exclude | Excluded | Exclude |
| LEP-252 | 2023 | Exclude | Exclude | Exclude | Exclude | 100% | Exclude | Excluded | Exclude |
| LEP-253 | 2023 | Exclude | Exclude | Exclude | Exclude | 100% | Exclude | Excluded | Exclude |
| LEP-254 | 2024 | Include | Exclude | Exclude | Exclude | 75% | Exclude | Excluded | Exclude |
| LEP-255 | 2024 | Exclude | Exclude | Exclude | Exclude | 100% | Exclude | Excluded | Exclude |
| LEP-256 | 2016 | Exclude | Exclude | Exclude | Exclude | 100% | Exclude | Excluded | Exclude |
| LEP-257 | 2024 | Exclude | Exclude | Exclude | Exclude | 100% | Exclude | Excluded | Exclude |
| LEP-258 | 2023 | Exclude | Exclude | Exclude | Exclude | 100% | Exclude | Excluded | Exclude |
| LEP-259 | 2023 | Include | Exclude | Include | Exclude | 50% | Exclude | Excluded | Exclude |
| LEP-260 | 2016 | Include | Exclude | Include | Exclude | 50% | Exclude | Excluded | Exclude |
| LEP-261 | 2020 | Exclude | Exclude | Exclude | Exclude | 100% | Exclude | Excluded | Exclude |
| LEP-262 | 2023 | Include | Exclude | Include | Exclude | 50% | Exclude | Excluded | Exclude |
| LEP-263 | 2019 | Exclude | Exclude | Exclude | Exclude | 100% | Exclude | Excluded | Exclude |
| LEP-264 | 2021 | Exclude | Exclude | Exclude | Exclude | 100% | Exclude | Excluded | Exclude |
| LEP-265 | 2020 | Include | Exclude | Exclude | Exclude | 75% | Exclude | Excluded | Exclude |
| LEP-266 | 2023 | Exclude | Exclude | Exclude | Exclude | 100% | Exclude | Excluded | Exclude |
| LEP-267 | 2019 | Exclude | Exclude | Exclude | Exclude | 100% | Exclude | Excluded | Exclude |
| LEP-268 | 2021 | Exclude | Exclude | Exclude | Exclude | 100% | Exclude | Excluded | Exclude |
| LEP-269 | 2017 | Include | Exclude | Include | Exclude | 50% | Exclude | Excluded | Exclude |
| LEP-270 | 2016 | Exclude | Exclude | Exclude | Exclude | 100% | Exclude | Excluded | Exclude |
| LEP-271 | 2020 | Exclude | Exclude | Exclude | Exclude | 100% | Exclude | Excluded | Exclude |
| LEP-272 | 2023 | Exclude | Exclude | Exclude | Exclude | 100% | Exclude | Excluded | Exclude |
| LEP-273 | 2019 | Exclude | Exclude | Exclude | Exclude | 100% | Exclude | Excluded | Exclude |
| LEP-274 | 2021 | Exclude | Exclude | Exclude | Exclude | 100% | Exclude | Excluded | Exclude |
| LEP-275 | 2024 | Include | Exclude | Exclude | Exclude | 75% | Exclude | Excluded | Exclude |
| LEP-276 | 2024 | Exclude | Exclude | Exclude | Exclude | 100% | Exclude | Excluded | Exclude |
| LEP-277 | 2016 | Exclude | Exclude | Exclude | Exclude | 100% | Exclude | Excluded | Exclude |
| LEP-278 | 2024 | Exclude | Exclude | Exclude | Exclude | 100% | Exclude | Excluded | Exclude |
| LEP-279 | 2024 | Exclude | Exclude | Exclude | Exclude | 100% | Exclude | Excluded | Exclude |
| LEP-280 | 2024 | Exclude | Exclude | Exclude | Exclude | 100% | Exclude | Excluded | Exclude |
| LEP-281 | 2016 | Include | Exclude | Exclude | Exclude | 75% | Exclude | Excluded | Exclude |
| LEP-282 | 2024 | Exclude | Exclude | Exclude | Exclude | 100% | Exclude | Excluded | Exclude |
| LEP-283 | 2023 | Include | Exclude | Include | Exclude | 50% | Exclude | Excluded | Exclude |
| LEP-284 | 2023 | Exclude | Exclude | Exclude | Exclude | 100% | Exclude | Excluded | Exclude |
| LEP-285 | 2016 | Exclude | Exclude | Exclude | Exclude | 100% | Exclude | Excluded | Exclude |
| LEP-286 | 2020 | Exclude | Exclude | Exclude | Exclude | 100% | Exclude | Excluded | Exclude |
| LEP-287 | 2024 | Exclude | Exclude | Exclude | Exclude | 100% | Exclude | Excluded | Exclude |
| LEP-288 | 2024 | Exclude | Exclude | Exclude | Exclude | 100% | Exclude | Excluded | Exclude |
| LEP-289 | 2016 | Include | Exclude | Exclude | Exclude | 75% | Exclude | Excluded | Exclude |
| LEP-290 | 2024 | Exclude | Exclude | Exclude | Exclude | 100% | Exclude | Excluded | Exclude |
| LEP-291 | 2023 | Include | Exclude | Include | Exclude | 50% | Exclude | Excluded | Exclude |
| LEP-292 | 2023 | Exclude | Exclude | Exclude | Exclude | 100% | Exclude | Excluded | Exclude |
| LEP-293 | 2016 | Exclude | Exclude | Exclude | Exclude | 100% | Exclude | Excluded | Exclude |
| LEP-294 | 2020 | Exclude | Exclude | Exclude | Exclude | 100% | Exclude | Excluded | Exclude |
| LEP-295 | 2023 | Exclude | Exclude | Exclude | Exclude | 100% | Exclude | Excluded | Exclude |
| LEP-296 | 2019 | Include | Exclude | Exclude | Exclude | 75% | Exclude | Excluded | Exclude |
| LEP-297 | 2024 | Exclude | Exclude | Exclude | Exclude | 100% | Exclude | Excluded | Exclude |
| LEP-298 | 2024 | Include | Exclude | Include | Exclude | 50% | Exclude | Excluded | Exclude |
| LEP-299 | 2016 | Exclude | Exclude | Exclude | Exclude | 100% | Exclude | Excluded | Exclude |
| LEP-300 | 2024 | Exclude | Exclude | Exclude | Exclude | 100% | Exclude | Excluded | Exclude |
| LEP-301 | 2023 | Exclude | Exclude | Exclude | Exclude | 100% | Exclude | Excluded | Exclude |
| LEP-302 | 2023 | Exclude | Exclude | Exclude | Exclude | 100% | Exclude | Excluded | Exclude |
| LEP-303 | 2016 | Exclude | Exclude | Exclude | Exclude | 100% | Exclude | Excluded | Exclude |
| LEP-304 | 2020 | Exclude | Exclude | Exclude | Exclude | 100% | Exclude | Excluded | Exclude |
| LEP-305 | 2023 | Include | Exclude | Exclude | Exclude | 75% | Exclude | Excluded | Exclude |
| LEP-306 | 2019 | Exclude | Exclude | Exclude | Exclude | 100% | Exclude | Excluded | Exclude |
| LEP-307 | 2021 | Exclude | Exclude | Exclude | Exclude | 100% | Exclude | Excluded | Exclude |
| LEP-308 | 2020 | Exclude | Exclude | Exclude | Exclude | 100% | Exclude | Excluded | Exclude |
| LEP-309 | 2023 | Exclude | Exclude | Exclude | Exclude | 100% | Exclude | Excluded | Exclude |
| LEP-310 | 2019 | Exclude | Exclude | Exclude | Exclude | 100% | Exclude | Excluded | Exclude |
| LEP-311 | 2021 | Include | Exclude | Include | Exclude | 50% | Exclude | Excluded | Exclude |
| LEP-312 | 2017 | Exclude | Exclude | Exclude | Exclude | 100% | Exclude | Excluded | Exclude |
| LEP-313 | 2016 | Exclude | Exclude | Exclude | Exclude | 100% | Exclude | Excluded | Exclude |
| LEP-314 | 2020 | Exclude | Exclude | Exclude | Exclude | 100% | Exclude | Excluded | Exclude |
| LEP-315 | 2023 | Exclude | Exclude | Exclude | Exclude | 100% | Exclude | Excluded | Exclude |
| LEP-316 | 2019 | Include | Exclude | Exclude | Exclude | 75% | Exclude | Excluded | Exclude |
| LEP-317 | 2021 | Exclude | Exclude | Exclude | Exclude | 100% | Exclude | Excluded | Exclude |
| LEP-318 | 2016 | Include | Include | Include | Include | 100% | Exclude | Excluded | Exclude |
| LEP-319 | 2023 | Include | Exclude | Include | Exclude | 50% | Exclude | Excluded | Exclude |
| LEP-320 | 2015 | Exclude | Exclude | Exclude | Exclude | 100% | Exclude | Excluded | Exclude |
| LEP-321 | 2016 | Exclude | Exclude | Exclude | Exclude | 100% | Exclude | Excluded | Exclude |
| LEP-322 | 2024 | Exclude | Exclude | Exclude | Exclude | 100% | Exclude | Excluded | Exclude |
| LEP-323 | 2024 | Include | Exclude | Exclude | Exclude | 75% | Exclude | Excluded | Exclude |
| LEP-324 | 2024 | Exclude | Exclude | Exclude | Exclude | 100% | Exclude | Excluded | Exclude |
| LEP-325 | 2024 | Include | Include | Include | Include | 100% | Exclude | Excluded | Exclude |
| LEP-326 | 2016 | Include | Include | Include | Include | 100% | Exclude | Excluded | Exclude |
| LEP-327 | 2024 | Exclude | Exclude | Exclude | Exclude | 100% | Exclude | Excluded | Exclude |
| LEP-328 | 2023 | Include | Exclude | Include | Exclude | 50% | Exclude | Excluded | Exclude |
| LEP-329 | 2023 | Exclude | Exclude | Exclude | Exclude | 100% | Exclude | Excluded | Exclude |
| LEP-330 | 2016 | Exclude | Exclude | Exclude | Exclude | 100% | Exclude | Excluded | Exclude |
| LEP-331 | 2020 | Exclude | Exclude | Exclude | Exclude | 100% | Exclude | Excluded | Exclude |
| LEP-332 | 2023 | Exclude | Exclude | Exclude | Exclude | 100% | Exclude | Excluded | Exclude |
| LEP-333 | 2019 | Exclude | Exclude | Exclude | Exclude | 100% | Exclude | Excluded | Exclude |
| LEP-334 | 2021 | Include | Exclude | Include | Exclude | 50% | Exclude | Excluded | Exclude |
| LEP-335 | 2020 | Exclude | Exclude | Exclude | Exclude | 100% | Exclude | Excluded | Exclude |
| LEP-336 | 2023 | Exclude | Exclude | Exclude | Exclude | 100% | Exclude | Excluded | Exclude |
| LEP-337 | 2019 | Exclude | Exclude | Exclude | Exclude | 100% | Exclude | Excluded | Exclude |
| LEP-338 | 2021 | Exclude | Exclude | Exclude | Exclude | 100% | Exclude | Excluded | Exclude |
| LEP-339 | 2017 | Include | Exclude | Exclude | Exclude | 75% | Exclude | Excluded | Exclude |
| LEP-340 | 2016 | Exclude | Exclude | Exclude | Exclude | 100% | Exclude | Excluded | Exclude |
| LEP-341 | 2020 | Exclude | Exclude | Exclude | Exclude | 100% | Exclude | Excluded | Exclude |
| LEP-342 | 2023 | Exclude | Exclude | Exclude | Exclude | 100% | Exclude | Excluded | Exclude |
| LEP-343 | 2019 | Exclude | Exclude | Exclude | Exclude | 100% | Exclude | Excluded | Exclude |
| LEP-344 | 2021 | Include | Include | Include | Include | 100% | Exclude | Excluded | Exclude |
| LEP-345 | 2016 | Exclude | Exclude | Exclude | Exclude | 100% | Exclude | Excluded | Exclude |
| LEP-346 | 2023 | Exclude | Exclude | Exclude | Exclude | 100% | Exclude | Excluded | Exclude |
| LEP-347 | 2015 | Include | Include | Include | Include | 100% | Exclude | Excluded | Exclude |
| LEP-348 | 2021 | Include | Exclude | Include | Exclude | 50% | Exclude | Excluded | Exclude |
| LEP-349 | 2023 | Exclude | Exclude | Exclude | Exclude | 100% | Exclude | Excluded | Exclude |
