# Supplementary material for: Machine Learning and Deep Learning Techniques for Prediction and Diagnosis of Leptospirosis: Systematic Literature Review
Source: JMIR Med Inform. 2025 May 29;13:e67859. doi: 10.2196/67859 (PMC12140502; doi:10.2196/67859)
Supplement: Multimedia Appendix 3 [file medinform-v13-e67859-s003.docx]

### Appendix 3: Quality assessment/risk of bias

| **Author** | **Participant Bias** | **Predictor Bias** | **Outcome Bias** | **Analysis Bias** | **Overall Bias Rating** | **Overall Applicability Rating** |
| --- | --- | --- | --- | --- | --- | --- |
| Douchet, Léa et al. | Low | Medium | Medium | Medium | Medium | High |
| Rahmat, Fariq et al. | Medium | Medium | Medium | Medium | Medium | High |
| Caicedo Torres, W. et al. | Medium | Medium | Low | Low | Medium | High |
| Nery, Nivison Ruy R. et al. | Medium | Medium | Low | Low | Medium | High |
| Shenoy, Shreelaxmi et al. | Medium | Medium | Low | Medium | Medium | High |
| Sonthayanon, Piengchan et al. | Medium | Medium | Low | Medium | Medium | High |
| Nery Jr, Nivison Ruy Rocha et al. | Medium | Medium | Low | Medium | Medium | High |
| Mayfield, Helen J. et al. | Medium | Medium | Low | Medium | Medium | High |
| Jayaramu, Veianthan et al. | Medium | Medium | Low | Medium | Medium | High |
| Galdino, Gabriela Studart et al. | Medium | Medium | Low | Medium | Medium | High |
| Ahangarcani, Mehrdad et al. | Medium | Medium | Low | Medium | Medium | High |
| Mohammadinia, Ali et al. | Medium | Medium | Low | Medium | Medium | High |
| Kulkarni, Apeksha et al. | Low | Low | Low | Low | Low | High |
| Lopez, Diego Montenegro et al. | Medium | Medium | Low | Medium | Medium | High |
| Douchet, Léa et al. | Medium | Medium | Medium | Medium | Medium | High |
| Thibeaux, R. et al. | Medium | Medium | Medium | Medium | Medium | High |
| Zhao, Jian et al. | High | Medium | Low | Low | Medium | High |
