# Supplementary material for: Machine Learning and Deep Learning Techniques for Prediction and Diagnosis of Leptospirosis: Systematic Literature Review
Source: JMIR Med Inform. 2025 May 29;13:e67859. doi: 10.2196/67859 (PMC12140502; doi:10.2196/67859)
Supplement: Multimedia Appendix 4 [file medinform-v13-e67859-s004.docx]

Appendix 4. Characteristics of included studies on machine learning and deep learning applications for leptospirosis prediction and diagnosis.

| **Author** | **Title** | **Publication Type** | **Publication Year** | **Country of Research** | **Source of Data** | **Type of Data (Public/Private)** | **Type of Data Used** | **Number of Sample** | **Objective** | **Research Focus** | **Data Collection Method** | **ML Algorithm** | **DL Algorithms** | **Model (Pre-trained/Scratch)** | **Transfer Learning (Yes/No)** | **Data Augmentation (Yes/No)** | **Validation Method** | **Model Evaluation Metric** | **Strong Prediction of Leptospirosis (%)** | **Main Outcome Metric** | **Task** | **Type of Classification** | **Limitations** |
| --- | --- | --- | --- | --- | --- | --- | --- | --- | --- | --- | --- | --- | --- | --- | --- | --- | --- | --- | --- | --- | --- | --- | --- |
| Douchet, Léa et al. | Unraveling the invisible leptospirosis in mainland Southeast Asia and its fate under climate change. | Journal Article | 2022 | Thailand, Myanmar, Cambodia, Vietnam, Laos | Thai surveillance system | Public | Environmental, spatial, climatic, topographic, land cover, soil variables | NS | To investigate the environmental factors driving leptospirosis and estimate its current and future burden in mainland Southeast Asia | Prediction | Surveillance data, climate projections | Support Vector Regression (SVR) | NS | Scratch | No | No | Leave-one-out cross-validation (LOO-CV) | Mean Squared Error (MSE) | Correlation: 88% | Predicted incidence of leptospirosis | Prediction | NA | Under- and over-reporting bias, exclusion of human behavior and socio-economic factors, regional extrapolation caution, aggregation of ecological variables |
| Rahmat, Fariq et al. | Exploratory Data Analysis and Artificial Neural Network for Prediction of Leptospirosis Occurrence in Seremban, Malaysia Based on Meteorological Data | Journal Article | 2020 | Malaysia | Meteorological data from the Malaysia Meteorological Department, rainfall data from the Department of Irrigation and Drainage, leptospirosis case data from the State Department of Health, Negeri Sembilan. | Public | Environmental, clinical (reported leptospirosis cases), spatial | 364 observations (weeks) | To analyze, capture, and predict the relation between leptospirosis occurrence and meteorological data using EDA and ANN. | Prediction | Retrospective data collection from government sources | NS | Artificial Neural Network (ANN) | Scratch | No | No | Split-validation (80% training, 15% testing, 5% validation) | Accuracy, sensitivity, specificity, ROC curve, AUC | accuracy 84%, sensitivity, 86.44%, and specificity 79.33%, | Accuracy | Classification | Binary (occurrence vs. non-occurrence of leptospirosis) | Confined sample to specific regions may not represent broader populations. |
| Caicedo Torres, W. et al. | Differential diagnosis of hemorrhagic fevers using ARTMAP and an Artificial Immune System | Journal Article | 2012 | Colombia | Medical charts from patients treated in the last 10 years at Napoleón Franco Pareja Children Hospital, Cartagena, Colombia | Private | Clinical, demographic | 136 patients | To perform differential diagnosis of Dengue Fever, Leptospirosis, and Malaria using neural networks | Diagnosis | Medical charts review | Adaptive Resonance Theory Map (ARTMAP), FuzzyARTMAP, ARTMAP-IC, Default ARTMAP2 | NS | Scratch | No | No | Stratified random sampling with proportional assignment, feature selection using CLONALG (Artificial Immune System) | Recall, Macro F1-Score | Validation Set Results (Recall): FuzzyARTMAP: 80% ARTMAP-IC: 100% DefaultARTMAP2: 100% Test Set Results (Recall):  FuzzyARTMAP: 80% ARTMAP-IC: 60% DefaultARTMAP2: 60% | Macro F1-Score, Recall | Classification | Multi-class | Limited sample size for Leptospirosis and Malaria and high variability in performance for less represented classes |
| Nery, Nivison Ruy R. et al. | Classification model analysis for the prediction of leptospirosis cases | Conference Paper | 2016 | Brazil and United States | Hospital records, Patient interviews, Medical charts, Laboratory test results | Private | Clinical data, Epidemiological data, Demographic data, Georeference data | 4,675 instances | To analyze classification models for predicting leptospirosis cases based on clinical and epidemiological data | Prediction | Active hospital surveillance, ambulatory monitoring, community cohort monitoring | Decision Tree (J48, REPTree), Classification Rules (JRip, OneR, PART, DecisionTable), Bayesian Classification (Naive Bayes) | NS | Scratch | No | No | Percentage Split (PS) 66%, Percentage Split (PS) 80%, Cross Validation (CV) with 10 Folds | Sensitivity, Specificity, Kappa statistic, True Positive Rate, False Positive Rate, F-Measure, Matthews Correlation Coefficient, ROC Area | JRip algorithm with 85% sensitivity and 81% specificity | Accuracy of the classification model | Classification | Binary | Missing data in attributes, Most attributes created in the last five years, causing many empty values, Focus on clinical and epidemiological data without corresponding laboratory data |
| Shenoy, Shreelaxmi et al. | Artificial intelligence in differentiating tropical infections: A step ahead | Journal Article | 2022 | India | Medical record department (retrospective data), Self-administered questionnaires (for need analysis) | Private | Clinical data, Laboratory data, Epidemiological data | 800 patients (200 in each group: dengue, malaria, leptospirosis, and scrub typhus) | To develop a clinician-assisted decision-making tool to differentiate common tropical infections | Diagnosis | Retrospective data collection from medical records, self-administered questionnaires for need analysis | Decision Tree, Random Forest, Multinomial Logistic Regression, AdaBoost | Neural Networks | Scratch | No | No | 10-fold Cross Validation, Percentage Split (66% and 80%) | Accuracy, True Positive Rate (Sensitivity/Recall), False Positive Rate, Precision (Positive Predictive Value), F-Measure, ROC Area | Leptospirosis vs. Others Test Method: Training Set Naïve Bayes:  TP Rate/Sensitivity/Recall: 85% FP Rate: 20% Precision/PPV: 86% F-Measure: 85% ROC Area: 91% K-NN:  TP Rate/Sensitivity/Recall: 87% FP Rate: 27% Precision/PPV: 87% F-Measure: 86% ROC Area: 94% Multi Layer Perceptron:  TP Rate/Sensitivity/Recall: 99% FP Rate: 0.91% Precision/PPV: 99% F-Measure: 99% ROC Area: 99% J48 Decision Tree:  TP Rate/Sensitivity/Recall: 91% FP Rate: 19% Precision/PPV: 91% F-Measure: 91% ROC Area: 92% Random Forest:  TP Rate/Sensitivity/Recall: 100% FP Rate: 0% Precision/PPV: 100% F-Measure: 100% ROC Area: 100% Multinomial Logistic Regression:  TP Rate/Sensitivity/Recall: 89% FP Rate: 20% Precision/PPV: 89.7% F-Measure: 89% ROC Area: 94% Adaboost:  TP Rate/Sensitivity/Recall: 87% FP Rate: 29% Precision/PPV: 87% F-Measure: 86% ROC Area: 90% Test Method: 10-fold cross validation Naïve Bayes:  TP Rate/Sensitivity/Recall: 84% FP Rate: 21% Precision/PPV: 85% F-Measure: 84% ROC Area: 90% K-NN:  TP Rate/Sensitivity/Recall: 84% FP Rate: 34% Precision/PPV: 83% F-Measure: 83% ROC Area: 88% Multi Layer Perceptron:  TP Rate/Sensitivity/Recall: 85% FP Rate: 27% Precision/PPV: 84% F-Measure: 84% ROC Area: 89% J48 Decision Tree:  TP Rate/Sensitivity/Recall: 85% FP Rate: 31% Precision/PPV: 84% F-Measure: 84% ROC Area: 82% Random Forest:  TP Rate/Sensitivity/Recall: 87% FP Rate: 30% Precision/PPV: 86% F-Measure: 86% ROC Area: 91% Multinomial Logistic Regression:  TP Rate/Sensitivity/Recall: 86% FP Rate: 25% Precision/PPV: 86% F-Measure: 86% ROC Area: 90% Adaboost:  TP Rate/Sensitivity/Recall: 85% FP Rate: 33% Precision/PPV: 85% F-Measure: 84% ROC Area: 87% Dengue vs. Leptospirosis Test Method: Training Set Naïve Bayes:  TP Rate/Sensitivity/Recall: 89% FP Rate: 10% Precision/PPV: 89% F-Measure: 89% ROC Area: 96% K-NN:  TP Rate/Sensitivity/Recall: 90% FP Rate: 9% Precision/PPV: 90% F-Measure: 90% ROC Area: 97% Multi Layer Perceptron:  TP Rate/Sensitivity/Recall: 99% FP Rate: 0% Precision/PPV: 99% F-Measure: 99% ROC Area: 99% J48 Decision Tree:  TP Rate/Sensitivity/Recall: 91% FP Rate: 9% Precision/PPV: 91% F-Measure: 91% ROC Area: 95% Random Forest:  TP Rate/Sensitivity/Recall: 100% FP Rate: 0% Precision/PPV: 100% F-Measure: 100% ROC Area: 100% Multinomial Logistic Regression:  TP Rate/Sensitivity/Recall: 94% FP Rate: 5% Precision/PPV: 94% F-Measure: 94% ROC Area: 98% Adaboost:  TP Rate/Sensitivity/Recall: 88% FP Rate: 12% Precision/PPV: 88% F-Measure: 88% ROC Area: 94% Testing Method: 10-fold cross validation Naïve Bayes:  TP Rate/Sensitivity/Recall: 87% FP Rate: 12% Precision/PPV: 87% F-Measure: 87% ROC Area: 95% K-NN:  TP Rate/Sensitivity/Recall: 86% FP Rate: 14% Precision/PPV: 86% F-Measure: 86% ROC Area: 92% Multi Layer Perceptron:  TP Rate/Sensitivity/Recall: 87% FP Rate: 12% Precision/PPV: 87% F-Measure: 87% ROC Area: 94% J48 Decision Tree:  TP Rate/Sensitivity/Recall: 82% FP Rate: 17% Precision/PPV: 82% F-Measure: 82% ROC Area: 86% Random Forest:  TP Rate/Sensitivity/Recall: 87% FP Rate: 12% Precision/PPV: 87% F-Measure: 87% ROC Area: 95% Multinomial Logistic Regression:  TP Rate/Sensitivity/Recall: 86% FP Rate: 13% Precision/PPV: 86% F-Measure: 86% ROC Area: 92% Adaboost:  TP Rate/Sensitivity/Recall: 84% FP Rate: 15% Precision/PPV: 84% F-Measure: 84% ROC Area: 92% | Accuracy of the classification model | Classification | Binary, Multi-class | Retrospective data collection leading to missing initial clinical parameters, Findings from single-centered data cannot be generalized globally, WEKA software limitations (does not provide true negative, false negative, and specificity) |
| Sonthayanon, Piengchan et al. | Whole cell matrix assisted laser desorption/ionization time-of-flight mass spectrometry (MALDI-TOF MS) for identification of Leptospira spp. in Thailand and Lao PDR | Journal Article | 2019 | Thailand and Laos | Mahidol Oxford Tropical Medicine Research Unit Data Access Committee | Private | Clinical data, Laboratory data | 116 Leptospira isolates including 15 reference strains and 101 clinical isolates, with validation on 97 clinical isolates | To use MALDI-TOF MS as a rapid and accurate tool for the identification of Leptospira spp. | Diagnosis | Retrospective data collection from clinical isolates and reference strains | Genetic Algorithm (GA), Quick Classifier (QC) | Supervised Neural Network (SNN) | Scratch | No | No | Profile matching to the MALDI-TOF MS database, Cross-validation | Recognition Capacity (RC), Cross Validation (CV), Area Under the Receiver Operating Characteristic (ROC) curve (AUC), Identification score | accuracy 98.9% | Identification score and classification accuracy | Classification | Multi-class (different Leptospira species) | Requirement of bacterial culture which is time-consuming, Lack of urine samples from leptospirosis patients for further validation, Detection limit and reproducibility issues for higher molecular weight proteins |
| Nery Jr, Nivison Ruy Rocha et al. | Prediction of leptospirosis cases using classification algorithms | Journal Article | 2017 | Brazil and USA | Gonçalo Moniz Institute (IGM) Oswaldo Cruz Foundation, Ministry of Health, Salvador, Brazil; Federal University of Bahia (UFBA), Salvador, Brazil; Yale School of Public Health, New Haven, CT, USA | Private | Clinical data, Epidemiological data | 4675 instances of suspected leptospirosis cases, including 2046 confirmed and 2629 unconfirmed cases | To analyze whether classification models applied to clinical and epidemiological patient data could accurately identify cases of leptospirosis | Diagnosis | Retrospective data collection from hospital and community cohort | JRIP, J48, REPTree, OneR, PART, DecisionTable (DT), Naive Bayes (NB) | NS | Scratch | No | No | Percentage Split (PS) 66% training, 34% testing; PS 80% training, 20% testing; Cross-validation (CV) with 10 folds | Sensitivity, Specificity, Accuracy, Kappa statistic, True positives, True negatives, False positives, False negatives, F-measure, Matthews correlation coefficient, Area Under the Receiver Operating Characteristic (ROC) curve | JRIP achieved 84% sensitivity and 81% specificity | Sensitivity and specificity | Classification | Binary classification (leptospirosis vs. non-leptospirosis); Multi-class classification (leptospirosis vs. dengue vs. other febrile illnesses) | The dataset had many missing values, particularly for attributes created in the last 5 years. Specificity decreased when new patient records from 2014-2016 were added to the test set. No validation with urine samples from leptospirosis patients. |
| Mayfield, Helen J. et al. | Predictive risk mapping of an environmentally-driven infectious disease using spatial Bayesian networks: A case study of leptospirosis in Fiji | Journal Article | 2018 | Fiji | Eco-epidemiological study, Government departments, Census information | Private | Spatially-explicit field data, Census data, Environmental and socio-demographic factors | 2,152 human participants in 82 villages | To examine drivers of leptospirosis transmission under different scenarios of environmental and livestock exposures using spatial Bayesian networks | Risk mapping and prediction | Field data collection, Surveys and questionnaires, Serological data, GPS coordinates | Bayesian networks (BNs), Tree augmented naïve (TAN) network | NS | Scratch | No | No | Area Under the Receiver Operating Curve (AUC), True Skill Statistic (TSS), Cross-validation with 50 trials | Sensitivity to findings analysis, AUC, TSS | The final model using a TAN network had a mean AUC of 0.89 and a mean TSS of 0.64 | Probability of above average seroprevalence | Predictive risk mapping | Binary | The dataset was limited to 82 villages, leading to a need to categorize variables into only two states. Spatial data for certain variables were not available. |
| Jayaramu, Veianthan et al. | Leptospirosis modelling using hydrometeorological indices and random forest machine learning | Journal Article | 2023 | Malaysia | Kelantan State Department of Health, Department of Irrigation and Drainage Malaysia (DID), Malaysian Meteorological Department (MetMalaysia) | Private | Weekly leptospirosis case data, Daily hydrometeorological data (rainfall, streamflow, water level, relative humidity, and temperature) | 517 weekly records of leptospirosis case numbers | To explore the use of a random forest classifier to analyze the relative importance of hydrometeorological indices in developing a leptospirosis model and to evaluate the performance of models based on the type of indices used | Predictive modeling of leptospirosis occurrence using hydrometeorological indices | Aggregation of weekly leptospirosis cases, Transformation of daily hydrometeorological data into 164 weekly average and extreme indices | Random forest classifier | NS | Scratch | No | No | 10-fold cross-validation | Prediction accuracy, Sensitivity, Specificity, Mean decrease Gini (MDG) score, Cross-correlation analysis, Receiver operating characteristic (ROC) analysis | Mixed models showed an improved prediction accuracy (71.7–82.6%); Extreme models were the most sensitive; Average models were the most specific | Prediction accuracy of leptospirosis occurrence (binary classification: high vs. low cases) | Predictive risk modeling | Binary classification | Spatial variability in the study area was not explicitly considered; Fixed thresholds may not represent the extreme limits of hydrometeorological events accurately. |
| Galdino, Gabriela Studart et al. | Development and validation of a simple machine learning tool to predict mortality in leptospirosis | Journal Article | 2023 | Brazil | Three tertiary reference hospitals in Fortaleza, state of Ceara, Brazil | Private | Admission data of leptospirosis patients | 295 leptospirosis patients | To develop and validate a machine learning tool to predict mortality in leptospirosis patients at hospital admission | Predictive modeling of mortality in leptospirosis patients | Retrospective collection from medical records | Lasso regression, Gradient boosting decision trees (xgBoost) | NS | Scratch | No | Yes | Internal validation cohort, Bootstrap resampling | Area under the curve of the receiver operating characteristic (AUC-ROC), Balanced accuracy, Sensitivity, Specificity | LeptoScore had an AUC-ROC of 0.776, QuickLepto had an AUC-ROC of 0.788 | Mortality prediction in leptospirosis patients (binary classification: death vs. survival) | Predictive risk modeling | Binary classification | Retrospective study design, Data collected over 14 years, Lack of external cohort validation |
| Ahangarcani, Mehrdad et al. | Predictive risk mapping of human leptospirosis using support vector machine classification and multilayer perceptron neural network | Journal Article | 2019 | Iran | Center for Disease Control and Prevention, Ministry of Health and Medical Education of Iran; Meteorological Organization of Iran; Digital Elevation Models derived from Shuttle Radar Topography Mission satellite images; Moderate Resolution Imaging Spectroradiometer satellite images; Statistical Center of Iran | Public and Private | Monthly reported cases of leptospirosis and their occurrence location at the district level, Climate and topographical data | 1,863 cases of leptospirosis | To generate predictive risk maps of leptospirosis using spatial statistics, environmental variables, and machine learning | Predictive modeling of leptospirosis distribution | Longitudinal study from January 2009 to December 2014 | Support Vector Machine (SVM), Multilayer Perceptron (MLP) neural network | Multilayer Perceptron (MLP) | Scratch | No | No | Cross-validation, Grid-search | Receiver Operating Characteristic (ROC) curve, Kappa coefficient | SVM model had an AUC of 0.8548 and Kappa coefficient of 85.13% for annual prediction; MLP model had an AUC of 0.8336 and Kappa coefficient of 83.64% for annual prediction; Both models showed high accuracy for monthly predictions with AUC values close to 0.88 | Predictive risk maps of leptospirosis incidence (annual and monthly) | Predictive risk mapping | Multi-class classification | Retrospective study design, Data collected over a limited geographical area, Lack of external validation in different geographic regions |
| Mohammadinia, Ali et al. | Prediction mapping of human leptospirosis using ANN, GWR, SVM and GLM approaches | Journal Article | 2019 | Iran | National Ministry of Health and Treatment of Iran (NMHT); National Centre of Statistics of Iran; Ministry of Health and Meteorology Agency of Iran | Public | Disease data (positive ELISA blood test results of patients), Climate data (temperature, humidity, precipitation), Topographic data (elevation), Vegetation data (NDVI from satellite images), Population data of rural districts | 1,186 positive cases of leptospirosis from 2009 to 2011 | To model and predict the spatial distribution of leptospirosis in Gilan Province using Geographically Weighted Regression (GWR), Generalized Linear Model (GLM), Support Vector Machine (SVM), and Artificial Neural Network (ANN). | Spatial modelling and prediction of leptospirosis distribution | Longitudinal study from 2009 to 2011, Data normalization and integration using ArcGIS 10.2 and Microsoft Excel 2010 | Support Vector Machine (SVM), Geographically Weighted Regression (GWR), Generalized Linear Model (GLM) | Artificial Neural Network (ANN) | Scratch | No | No | Cross-validation, Leave-one-out cross-validation for SVM | Mean Square Error (MSE), Mean Absolute Error (MAE), Mean Relative Error (MRE), R-squared (R²) | GWR model: MSE = 0.050, MAE = 0.012, MRE = 0.011, R² = 0.85; SVM model: MSE = 0.137, MAE = 0.063, MRE = 0.018, R² = 0.80; GLM model: MSE = 0.118, MAE = 0.052, MRE = 0.017, R² = 0.78; ANN model: MSE = 0.137, MAE = 0.063, MRE = 0.018, R² = 0.75 | Predictive risk maps of leptospirosis incidence | Predictive risk mapping | Multi-class classification | Data limited to rural districts and not specific locations like paddy fields, Models do not consider socio-epidemiologic parameters, Need for further validation at different scales |
| Kulkarni, Apeksha et al. | Spirochaeta Bacteria Detection Using an Effective Semantic Segmentation Technique | Conference Paper | 2022 | India | Kaggle (online data repository) | Public | Microscopy images of blood samples, Manually annotated masks of the images | 366 images | To develop a deep learning-based method for detecting Spirochaete bacteria in blood using semantic segmentation techniques. | Diagnosis | Black Field Microscopy images with dimensions 256x256, Manual annotation of masks for semantic segmentation | NS | U-Net Convolutional Neural Network (CNN) | Scratch | No | Yes | Split-validation (80% for training and 20% for testing) | Accuracy, Validation accuracy, Training accuracy | accuracy of 98.02%. | Predictive accuracy of Spirochaete bacteria detection | Semantic segmentation | Multiclass classification (differentiate between bacteria, red blood cells, and background) | Limited to Spirochaete bacteria detection, Model faces challenges in handling unbalanced datasets, Specific to the dataset used; performance may vary with different datasets |
| Lopez, Diego Montenegro et al. | Evaluating the Surveillance System for Spotted Fever in Brazil Using Machine-Learning Techniques | Journal Article | 2017 | Brazil | Disease Notification Information System (SINAN), Secretaria de Estado de Saúde do Rio de Janeiro (SES/RJ) | Public | Epidemiological data, Clinical data, Environmental data | 890 reported cases of spotted fever | To analyze the performance of the Brazilian spotted fever (SF) surveillance system in diagnosing and confirming suspected cases in the state of Rio de Janeiro from 2007 to July 2016 using machine-learning techniques. | Diagnosis and prediction of disease occurrence | Notifications of suspected cases reported to SINAN, Data from Secretaria de Estado de Saúde do Rio de Janeiro (SES/RJ) | Probabilistic Neural Networks (PNN), Decision Trees (Best First Decision Tree, Decision Stump, Functional Tree, J48, Logistic Model Trees, Reduced-Error Pruning Tree, Simple Classification and Regression) | NS | Scratch | No | No | k-fold cross-validation | Accuracy, Kappa coefficient, Decision rules | Best First Decision Tree: Correct instances 67.1%, Kappa 0.2935. J48: Correct instances 70.5%, Kappa 0.3648. Reduced-Error Pruning Tree: Correct instances 68.2%, Kappa 0.3159. | Accuracy of the probabilistic neural network: 38.2%, Performance of decision trees with clinical and epidemiological variables | Classification | Multi-class classification | Low quality of data in SINAN, High proportion of missing or improperly recorded data fields, Neural network had poor performance due to insufficient training data |
| Douchet, Léa et al. | Climate-driven models of leptospirosis dynamics in tropical islands from three oceanic basins | Journal Article | 2024 | Multiple tropical islands from three oceanic basins: Guadeloupe, Reunion Island, Fiji, Futuna, New Caledonia, Tahiti | Leptospirosis surveillance data from seven tropical islands | Public | Surveillance data, Environmental data (precipitation and temperature) | Monthly records from seven tropical islands | To model leptospirosis seasonality and outbreaks in tropical islands based on precipitation and temperature indicators. | Prediction and analysis of leptospirosis dynamics | Leptospirosis data collected from surveillance records, Climate data retrieved from satellite images | Support Vector Regression (SVR) | NS | Scratch | No | No | Leave-Island-Out Cross-Validation (LIO CV) for seasonal model, Leave-Year-Out Cross-Validation (LYO CV) for inter-annual model | Mean Absolute Error (MAE), ΔMAE (percentage improvement in MAE), R² (correlation coefficient) | Mean Absolute Error (MAE): 0.87 R² values: Above 0.68 | Seasonal dynamics accuracy with a global MAE of 0.87, R² above 0.68 for all islands in seasonal model | Prediction and segmentation of leptospirosis seasonality and outbreaks | NA | Underestimation of peaks in incidence, Differences in surveillance systems and data quality among islands, Lack of inclusion of environmental indicators such as water pH, soil type, and land use, Influence of local specificities and lifestyles on disease dynamics not fully captured |
| Thibeaux, R. et al. | Rainfall-driven resuspension of pathogenic Leptospira in a leptospirosis hotspot | Journal Article | 2024 | New Caledonia | Field data collected from a 3 km² watershed in New Caledonia | Private | Hydrological variables, Pathogenic Leptospira concentrations in water samples | 226 water samples | To analyze the concentration of pathogenic Leptospira as a function of hydrological variables in a leptospirosis hotspot and to evaluate the resuspension dynamics of pathogenic Leptospira during rainfall events. | Prediction and analysis of pathogenic Leptospira concentrations | Water sample collection during and after rainfall events, Measurement of hydrological variables (rainfall, water level, soil moisture, suspended matter concentration) | NS | Feedforward neural network | Scratch | No | No | Training, validation, and testing data splits (2/3 for training, 1/6 for validation, 1/6 for testing) | Root Mean Square Error (RMSE), Explained variance (Nash-Sutcliffe criterion) | The explained variance (R²): 75% | Explained variance of 75% for the machine learning model | Prediction of pathogenic Leptospira concentrations | NA | Small dataset, Complexity and variability of environmental factors, Influence of invasive animal species not fully accounted for, Study focused on a small watershed; larger studies needed for validation |
| Zhao, Jian et al. | Mapping risk of leptospirosis in China using environmental and socioeconomic data | Journal Article | 2016 | China | China’s web-based infectious disease reporting system, maintained by Chinese Center for Disease Control and Prevention | Public | Environmental, socioeconomic, spatial | 2741 leptospirosis cases (2129 spatially unique occurrence points) | Identify environmental and socioeconomic factors associated with leptospirosis and predict potential risk areas using predictive models | Prediction | Geo-coding of leptospirosis incidence data from China CDC's database, extraction of environmental and socioeconomic variables | Logistic Regression, Maxent Model (Maximum Entropy) | NS | Scratch | No | No | Receiver Operating Characteristic (ROC) analysis, Area Under the Curve (AUC) | AUC value (Logistic Regression: 0.95, Maxent: 0.96) | logistic regression model with AUC value of 0.95 and and Maxent model WITH auc VALUE OF 0.96 | Proportion of risk area, AUC value | Classification | Binary (presence or absence of leptospirosis) | Coarse spatial resolution of river density data, GDP data not at fine spatial resolution, Lack of sanitation-related variables and fine-scale socioeconomic data, Need for more temporal and varied data for better validation |
