# Supplementary material for: Machine Learning and Deep Learning Techniques for Prediction and Diagnosis of Leptospirosis: Systematic Literature Review
Source: JMIR Med Inform. 2025 May 29;13:e67859. doi: 10.2196/67859 (PMC12140502; doi:10.2196/67859)
Supplement: Checklist 1 [file medinform-v13-e67859-s005.docx]

Supplementary Table 5: PRISMA 2020 Checklist for Systematic Reviews

| Section and Topic | Item # | Checklist Item | Location in the Paper |
| --- | --- | --- | --- |
| TITLE |  |  |  |
| Title | 1 | Identify the report as a systematic review. | 1 |
| ABSTRACT |  |  |  |
| Abstract | 2 | Provide a structured summary including objectives, methods, results, and conclusions. | 1 |
| INTRODUCTION |  |  |  |
| Rational | 3 | Describe the rationale for the review in the context of existing knowledge. | 2 |
| Objectives | 4 | Provide an explicit statement of the objectives and questions addressed. | 3-4 |
| METHODS |  |  |  |
| Information sources | 5 | List all databases, registers, websites, and sources searched, including search dates. | 4 |
| Search strategy | 6 | Present detailed search strategies for each database and include filters and limits used. | Appendix 1 |
| Eligibility criteria | 7 | Specify inclusion and exclusion criteria for the review. | 4,5 |
| Selection process | 8 | Specify methods for selecting studies, including reviewer roles and tools used. | 5 |
| Risk of bias assessment | 9 | Specify risk-of-bias assessment tools and processes. | 5 |
| Data collection process | 10 | Describe data extraction processes, reviewer roles, and automation tools if applicable. | 6 |
| Data items | 11a | Define outcomes and variables sought in the data. | 6 |
|  | 11b | List and define all other variables for which data were sought (e.g. participant and intervention characteristics, funding sources). Describe any assumptions made about any missing or unclear information. | 6, Appendix 4 |
| Effect measures | 12 | Describe the effect measures for outcomes. | 6 |
| Synthesis methods | 13a | Describe the processes used to decide which studies were eligible for each synthesis (e.g. tabulating the study intervention characteristics and comparing against the planned groups for each synthesis (item #5)). | 5,6 |
|  | 13b | Describe any methods required to prepare the data for presentation or synthesis, such as handling of missing summary statistics, or data conversions. | 5,6 |
|  | 13c | Describe any methods used to tabulate or visually display results of individual studies and syntheses. | 5,6 |
|  | 13d | Describe any methods used to synthesize results and provide a rationale for the choice(s). If meta-analysis was performed, describe the model(s), method(s) to identify the presence and extent of statistical heterogeneity, and software package(s) used. | 5,6 |
|  | 13e | Describe any methods used to explore possible causes of heterogeneity among study results (e.g. subgroup analysis, meta-regression). | 6 |
|  | 13f | Describe any sensitivity analyses conducted to assess robustness of the synthesized results | 6 |
| Reporting bias assessment | 14 | Describe any methods used to assess risk of bias due to missing results in a synthesis (arising from reporting biases). | 5,6 |
| Certainty assessment | 15 | Describe any methods used to assess certainty (or confidence) in the body of evidence for an outcome. | N/A |
| RESULTS |  |  |  |
| Study selection | 16a | Summarize the search results and selection process, preferably with a flow diagram. | 6-8 |
|  | 16b | Cite studies that might appear to meet the inclusion criteria, but which were excluded, and explain why they were excluded. | 6-8 |
| Study characteristics | 17 | Provide details on included studies and their characteristics. | 6,7, Appendix 4 |
| Risk of bias in studies | 18 | Report assessments of risk of bias for included studies. | 9, Appendix 3 |
| Results of individual studies | 19 | Present results for each outcome using structured tables or plots. | 7-16 |
| Results of syntheses | 20a | For each synthesis, briefly summarize the characteristics and risk of bias among contributing studies | 7-16, Appendix 3,4 |
|  | 20b | Present results of all statistical syntheses conducted. If meta-analysis was done, present for each the summary estimate and its precision (e.g. confidence/credible interval) and measures of statistical heterogeneity. If comparing groups, describe the direction of the effect. | 7-17 |
|  | 20c | Present results of all investigations of possible causes of heterogeneity among study results | 6-17 |
|  | 20d | Present results of all sensitivity analyses conducted to assess the robustness of the synthesized results. | 6-17, Appendix 3 |
| Reporting biases | 21 | Present risk of bias assessments due to missing results. | 9, Appendix 3 |
| Certainty of evidence | 22 | Assess confidence in evidence for each outcome. | 6-17 |
| DISCUSSION |  |  |  |
| Discussion | 23a | Provide general interpretation, limitations, and implications of results. | 18-20 |
|  | 23b | Discuss any limitations of the evidence included in the review | 18-20 |
|  | 23c | Discuss any limitations of the review processes used. | 18-20 |
|  | 23d | Discuss implications of the results for practice, policy, and future research | 18-20 |
| OTHER INFORMATION |  |  |  |
| Registration and protocol | 24a | Provide registration information for the review, including register name and registration number, or state that the review was not registered | N/A |
|  | 24b | Indicate where the review protocol can be accessed, or state that a protocol was not prepared. | N/A |
|  | 24c | Describe and explain any amendments to information provided at registration or in the protocol. | N/A |
| Support | 25 | Describe sources of financial or non-financial support for the review, and the role of the funders or sponsors in the review | N/A |
| Competing interest | 26 | Declare any competing interests of review authors. | N/A |
| Availability of data, code and other materials | 27 | Report which of the following are publicly available and where they can be found: template data collection forms; data extracted from included studies; data used for all analyses; analytic code; any other materials used in the review. | Appendix 1,2,3,4 |

From: Page MJ, McKenzie JE, Bossuyt PM, Boutron I, Hoffmann TC, Mulrow CD, et al. The PRISMA 2020 statement: an updated guideline for reporting systematic reviews. BMJ 2021;372:n71. doi: 10.1136/bmj.n71 For more information, visit: <http://www.prisma-statement.org/>
